# Improving the Postharvest Storage and Quality of Tomatoes (*Solanum lycopersicum* L.) Using Biochar Derived from Spent Tea Leaves

**DOI:** 10.3390/molecules31172982

**Published:** 2026-08-26

**Authors:** Aneta Saletnik, Dorota Grabek-Lejko, Czesław Puchalski, Bogdan Saletnik

**Affiliations:** Department of Bioenergetics, Food Analysis and Microbiology, Institute of Food Technology and Nutrition, Faculty of Technology and Life Sciences, University of Rzeszow, 2D Ćwiklińskiej Street, 35-601 Rzeszów, Poland; dgrabek@ur.edu.pl (D.G.-L.); cpuchalski@ur.edu.pl (C.P.); bsaletnik@ur.edu.pl (B.S.)

**Keywords:** tea-waste biochar, ethylene scavenging, tomato storage, postharvest horticulture, microbial quality, antioxidant activity, waste valorization, circular economy, Sustainable Development Goal 12

## Abstract

Ethylene accumulation accelerates the ripening of climacteric fruits and may impair their postharvest quality. This study evaluated biochar produced from spent tea leaves and enclosed in cellulose sachets as a material for reducing ethylene concentration and preserving tomato quality during storage. The biochar was produced by pyrolyzing spent black tea leaves at 400 °C for 10 min. Tomatoes were stored for 18 days at 21 °C in sealed glass chambers containing sachets with 1, 2, 3, 4, 5, 7.5, or 10 g of biochar, with a treatment without biochar serving as the control. After 6, 12, and 18 days, ethylene concentration, microbiological quality, and selected physicochemical and antioxidant properties were evaluated. Biochar reduced ethylene accumulation in a mass-dependent manner, with the most pronounced effects generally observed in the 5–10 g range. The 10 g treatment reduced ethylene concentration by 41.1%, 37.8%, and 40.3% relative to the control after 6, 12, and 18 days, respectively. The lowest counts of aerobic mesophilic microorganisms, yeasts, and molds were generally observed in the 7.5 and 10 g treatments. The application of at least 5 g of biochar most effectively limited firmness loss; after 18 days, firmness ranged from 1.77 to 1.80 kgf in the 5–10 g treatments, compared with 1.00 kgf in the control. Higher biochar masses also favored the retention of titratable acidity, total phenolic content, and antioxidant activity measured using the ABTS and FRAP assays. Overall, the beneficial effects on postharvest storage conditions and tomato quality became more evident with increasing biochar mass, particularly between 5 and 10 g. Sachets containing 7.5–10 g of spent-tea-leaf biochar showed the greatest potential as a simple passive system for controlling ethylene and preserving tomato quality. This approach combines waste valorization with the potential reduction in postharvest losses, supporting circular economy principles and Sustainable Development Goal 12 (SDG 12).

## 1. Introduction

Fruit ripening involves a series of genetically programmed modifications in biochemical, physiological, textural, sensory, and organoleptic characteristics. It is a highly organized and irreversible phenomenon [1,2]. Fruit maturity and senescence depend on ethylene evolution [3,4]. The phytohormone ethylene is produced at the onset of fruit ripening and regulates genes involved in the production of various ripening enzymes [2]. Thus, ethylene is responsible for numerous physiological processes in plants [3,5]. These include the ripening of fruits and vegetables, seed germination, cell elongation, defense against pathogens, flowering, dormancy, senescence, geotropism, and responses to external stress factors [6,7,8,9,10]. Ethylene was identified at the beginning of the 20th century as the first plant hormone. In 1901, Neljubov demonstrated that this hormone affected the growth of pea seedlings. Ethylene biosynthesis was elucidated and described in the 1970s, whereas the mechanisms of ethylene signaling were identified toward the end of the 1990s [11]. Many ethylene-mediated effects are required to initiate ripening in numerous horticultural products, such as color development in citrus fruits, tomatoes, and bananas. Ethylene production may be induced or stimulated when plant tissues are damaged [3,12]. Furthermore, ethylene is a highly reactive compound that can undergo reactions such as chemical cleavage, modification, adsorption, and absorption [5]. Continuous respiration and ethylene emission are associated with losses in the nutritional and sensory quality of food and with a shorter postharvest shelf life [13,14]. Therefore, ethylene release can be used as an indicator of fruit maturity [4]. Huang et al. (2022) demonstrated that the intensity of ethylene signaling during fruit ripening is closely associated with the progression toward full maturity, thereby confirming the key role of ethylene [8]. Each fruit has specific requirements regarding ethylene exposure, depending on the species, cultivar, stage of ripening, and ripening behavior (climacteric or nonclimacteric). Fresh produce releases different concentrations of ethylene, and sensitivity to ethylene exposure also varies among types of perishable products. Climacteric and nonclimacteric fruits differ primarily in their respiratory patterns and responses to ethylene. Climacteric fruits, including tomatoes, can ripen after harvest and exhibit a sharp increase in respiration and autocatalytic ethylene production. Nonclimacteric fruits, such as strawberries and grapes, do not show a comparable climacteric rise, and their response to ethylene is weaker [1,8,15]. These differences in ethylene production and sensitivity are particularly important during postharvest storage, when ethylene accumulation may directly influence the rate of ripening and quality deterioration.

As a gaseous hormone, ethylene can alter the physicochemical stability of fruits and vegetables at concentrations of 10–100 nL L^−1^ [16,17], leading to overripening, senescence, loss of texture, microbial attack, a shorter shelf life, and other related problems during fruit storage and transportation [15]. The adverse effects of ethylene on fresh fruits, vegetables, and ornamental plants can cause substantial product losses, reportedly reaching up to 80% [18,19]. Even small amounts of ethylene during transportation and storage have been observed to accelerate quality deterioration in fresh fruits, vegetables, and ornamental plants [20,21]. Symptoms associated with a short shelf life include decay, weight loss, deterioration in appearance and texture, and reduced nutritional quality [15]. These changes are regulated by cell-wall-degrading enzymes, such as cellulases and pectinases, and by pathogenesis-related proteins involved in tissue softening and degradation, as well as changes in color, flavor, and aroma. Inhibiting ethylene action can provide significant commercial benefits during the storage of ethylene-sensitive perishable products [1]. Food waste remains a major global problem, with fruits and vegetables accounting for a substantial proportion of discarded food [6,18]. Fruits are rich in vitamins, inorganic salts, dietary fiber, and other nutrients that are beneficial to human health. Effective regulation of ethylene during fruit transportation and storage is therefore essential to address the global challenge of postharvest fruit losses by limiting decay [3,18,19]. It is important to recognize that once fruit ripening approaches the climacteric stage, reducing the external ethylene concentration cannot reverse the ripening process. However, during the early stage of ripening, when the internal ethylene concentration is still very low, inhibiting ethylene production and/or removing ethylene may slow the ripening of climacteric perishable products [15]. Limiting this unavoidable risk is therefore necessary. Ethylene accumulation in packaging systems should be reduced to extend shelf life and maintain the visual and organoleptic quality of perishable products [1,19]. Consequently, effective management of ethylene in the postharvest environment is a key element of strategies aimed at slowing ripening and maintaining fruit quality during storage and transportation. The scientific literature describes several approaches for controlling ethylene concentrations. In fruit storage environments, modified-atmosphere preservation [9,22] and ethylene scavengers [7,23] are among the principal methods used for this purpose. However, modified-atmosphere preservation can be costly and time-consuming, may offer limited flexibility, and can adversely affect fruit appearance and flavor. Ethylene removal by scavengers is driven by van der Waals forces between adsorbent and adsorbate molecules. Adsorption, a surface phenomenon, depends on several parameters, including adsorbent concentration, temperature, gas composition, and relative humidity. Ethylene can be adsorbed using activated carbon, crystalline aluminosilicates, bentonite, alumina, fuller’s earth, brick dust, silica gel, and clay-based materials such as cristobalite and zeolite [6]. Although ethylene scavengers such as zeolites, activated carbon, porous metal oxides, silica gel, halloysite nanotubes, and montmorillonite have been reported to effectively remove ethylene released from fruit [24], they may have disadvantages, including incomplete degradation of ethylene adsorbed on their surfaces and within internal pores, ethylene desorption, and the generation of harmful residues [25,26]. Therefore, developing a highly efficient, safe, and sustainable ethylene scavenger that does not adversely affect the physicochemical or physiological properties of fruit is important for reducing fruit losses [6]. In this context, biochar represents a promising alternative because its porous carbonaceous structure may provide adsorption capacity while allowing the use of renewable or waste-derived feedstocks.

Biochar is a carbon-rich product obtained by heating organic matter under oxygen-limited [27] or anaerobic conditions [28]. It is regarded as an innovative material with numerous potential benefits, including improved soil water retention and fertility, mitigation of greenhouse gas emissions, carbon sequestration, reduction in acidity and salinity, and pathogen removal [29,30]. Biochar has a complex porous structure [31]. During pyrolysis, water is removed and volatile components are released from the carbon matrix, contributing to pore formation and the development of the primary pore structure. These pores are classified into three groups: micropores (<2 nm), mesopores (2–50 nm), and macropores (>50 nm) [32]. Porosity is a key factor in the use of biochar for adsorption purposes [33]. The porous nature of biochar increases its surface area [32], which is an important attribute in chemical reactions, adsorption, and interactions with other substances. At the microscopic level, biochar consists of a mixture of amorphous carbon, microcrystalline structures, and residual organic matter [34]. These structures contribute to its physical properties and reactivity. Yaashikaa et al. [35] reported that the biochar surface may contain functional groups, including hydroxyl, carboxyl, and phenolic groups, which are responsible for various chemical behaviors and interactions and may enhance sorption properties. Biochar is an adsorbent with many promising applications. Its high environmental stability, hydrophobicity, and substantial ion-exchange capacity, resulting from its developed porous structure, surface functional groups, and high specific surface area, make it a distinctive adsorbent for binding environmental contaminants [36,37].

These characteristics suggest that waste-derived biochar may also be suitable for applications beyond environmental remediation, including the control of gaseous compounds in postharvest storage systems. An adsorbent intended for postharvest applications should be produced from safe and sustainable feedstocks and should possess a developed porous structure that promotes effective ethylene capture. Biochar may meet these requirements because of its biocompatibility and sorption properties associated with pores and surface functional groups. In the present study, biochar derived from spent tea leaves was proposed as a natural, environmentally friendly, and potentially safe ethylene scavenger. This solution is consistent with circular economy principles because it enables the valorization of food-processing waste and may simultaneously contribute to reducing postharvest losses by controlling ethylene concentrations during fruit storage. Such an approach is consistent with Sustainable Development Goal 12 (Responsible Consumption and Production), particularly Target 12.3, which aims to reduce food losses along production and supply chains, including postharvest losses, and Target 12.5, which focuses on reducing waste generation through prevention, reduction, recycling, and reuse.

The available literature data indicate that biologically derived materials with a developed porous structure may effectively support ethylene control; however, the use of biochar derived from spent tea leaves for the postharvest protection of tomatoes remains insufficiently investigated. The novelty of this study lies in the simultaneous evaluation of the material’s ability to reduce ethylene concentration and its effects on the physicochemical and quality attributes of tomatoes during storage. The significance of the study derives from the possibility of developing a simple, inexpensive, waste-based solution that may slow ripening, limit quality losses, and extend the shelf life of climacteric fruit. Accordingly, this study addressed three principal research questions: (1) whether spent-tea-leaf biochar enclosed in cellulose sachets can reduce ethylene accumulation during tomato storage; (2) whether the effectiveness of this treatment depends on the mass of biochar applied; and (3) whether reduced ethylene accumulation is accompanied by improved preservation of microbiological, physicochemical, and antioxidant quality attributes. Therefore, the aim of this study was to evaluate the effects of sachets containing different masses of spent-tea-leaf biochar on ethylene concentration and tomato quality over successive storage periods.

## 2. Results and Discussion

### 2.1. Ethylene Concentration

**Figure 1:**
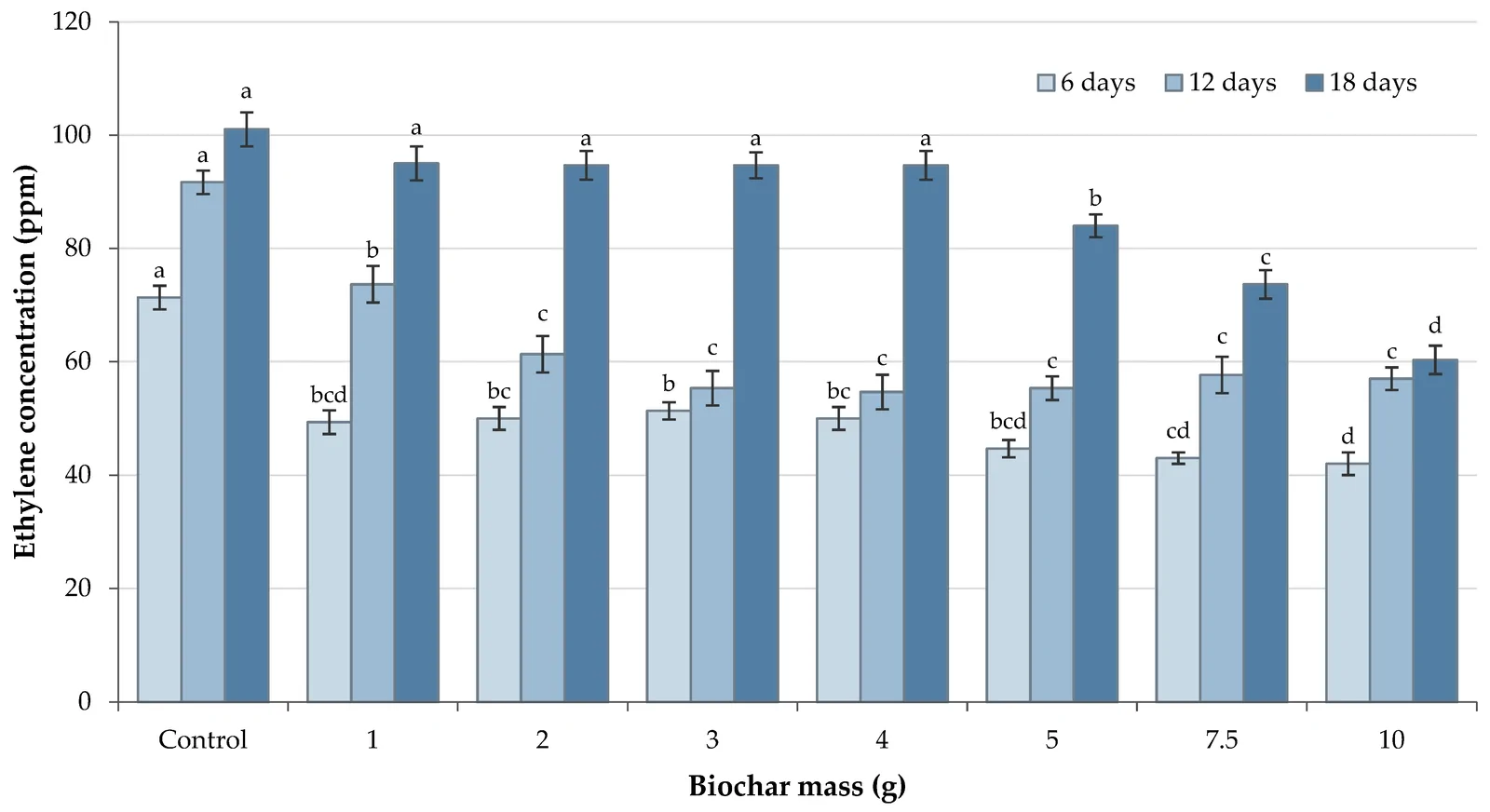
Ethylene concentration in the atmosphere of storage cloches containing tomatoes and different amounts of tea-waste biochar after 6, 12, and 18 days of storage. Different lowercase letters indicate statistically significant differences among treatments within the same storage time according to Tukey’s test (*p* < 0.05).

Ethylene concentration increased with storage time in all treatments. In the control, the mean concentration increased from 71.30 ppm after 6 days to 101.00 ppm after 18 days. Biochar reduced ethylene accumulation, and the magnitude of this effect generally increased with the mass applied. The strongest effect was observed for the 10 g treatment, in which ethylene concentrations were 42.00, 57.00, and 60.30 ppm after 6, 12, and 18 days, respectively. These values were 41.10%, 37.80%, and 40.30% lower than those in the control, and the differences were statistically significant at all storage times (*p* < 0.05). Treatments containing 5 and 7.5 g of biochar also reduced ethylene accumulation, particularly at the later storage stages. After 18 days, ethylene concentrations were 84.00 and 73.70 ppm in the 5 and 7.5 g treatments, respectively, whereas the values recorded for the 1–4 g treatments remained close to those of the control. Ethylene concentrations as a function of storage time and biochar mass are presented in Figure 1. The relatively high ethylene concentrations observed in the present study should be interpreted in the context of the experimental system. The tomatoes were stored in tightly sealed 5.6 dm^3^ glass chambers containing 1 kg of fruit, with no continuous ventilation or gas exchange. Consequently, ethylene released by the fruit accumulated progressively in the enclosed chamber atmosphere, resulting in concentrations that may be higher than those typically observed under ventilated commercial storage conditions. The use of fully ripe red-stage tomatoes was intended to reflect a practical consumer-oriented storage scenario. Therefore, the observed effects should be interpreted mainly in terms of limiting further ethylene accumulation and quality deterioration in already-ripe fruit, while the response may differ at earlier maturity stages.

The results indicate that biochar derived from spent tea leaves effectively limited ethylene accumulation in the cloche atmosphere, and that this effect increased with the mass of biochar applied. This relationship was particularly evident at the later storage times, when differences between the control and treatments containing greater amounts of biochar became more pronounced. The observed effect may be associated with the sorption properties generally reported for carbonaceous materials. Limiting ethylene accumulation in the storage environment may be important for slowing the ripening and senescence of climacteric fruit. The increase in ethylene concentration with storage time is consistent with the findings of Nurjanah et al. [38] and Rahman et al. [39], who observed increased ethylene production during the storage of Barangan bananas. Various ethylene-absorbing materials used to preserve the postharvest quality of fruit have also been described in the literature. Kaewklin et al. developed chitosan films containing nano-TiO_2_ that limited ethylene accumulation and extended the storage life of cherry tomatoes [40]. Tas et al. demonstrated that halloysite nanotubes with a high ethylene adsorption capacity delayed tomato softening and senescence [41]. Bailén et al. evaluated polypropylene packages containing sachets with 5 g of granular activated carbon and showed that the adsorbent delayed changes in tomato color and firmness and limited quality deterioration during storage [42]. Similarly, Oladipo et al. emphasized that postharvest strategies capable of modulating ethylene biosynthesis or limiting its effects may delay tomato ripening and contribute to the preservation of firmness, color, and antioxidant properties during storage [43]. The present results are therefore consistent with previous reports indicating that porous carbonaceous materials can limit ethylene accumulation in the storage environment. Biochar derived from spent tea leaves may thus represent a promising material for supporting the preservation of tomato quality during storage. The observed effects may be interpreted as a sequence of interconnected processes initiated by the adsorption of ethylene from the storage atmosphere. The porous structure and surface functional groups of biochar provide potential sites for interactions with gaseous compounds and may therefore contribute to limiting ethylene accumulation [35,36,37]. Reduced ethylene availability may, in turn, slow ethylene-mediated ripening and senescence processes, which are associated with progressive tissue softening and deterioration of postharvest quality [40,41,42,43]. This mechanism may help explain the better retention of firmness observed in treatments containing higher biochar masses. The better preservation of titratable acidity observed in these treatments may likewise reflect a slower progression of ripening and associated metabolic changes. These effects should primarily be interpreted as indirect consequences of modifying the storage atmosphere rather than as evidence of a direct physiological action of biochar on the fruit, because the biochar was enclosed in cellulose sachets and did not directly contact the tomatoes.

### 2.2. Microbiological Quality

**Figure 2:**
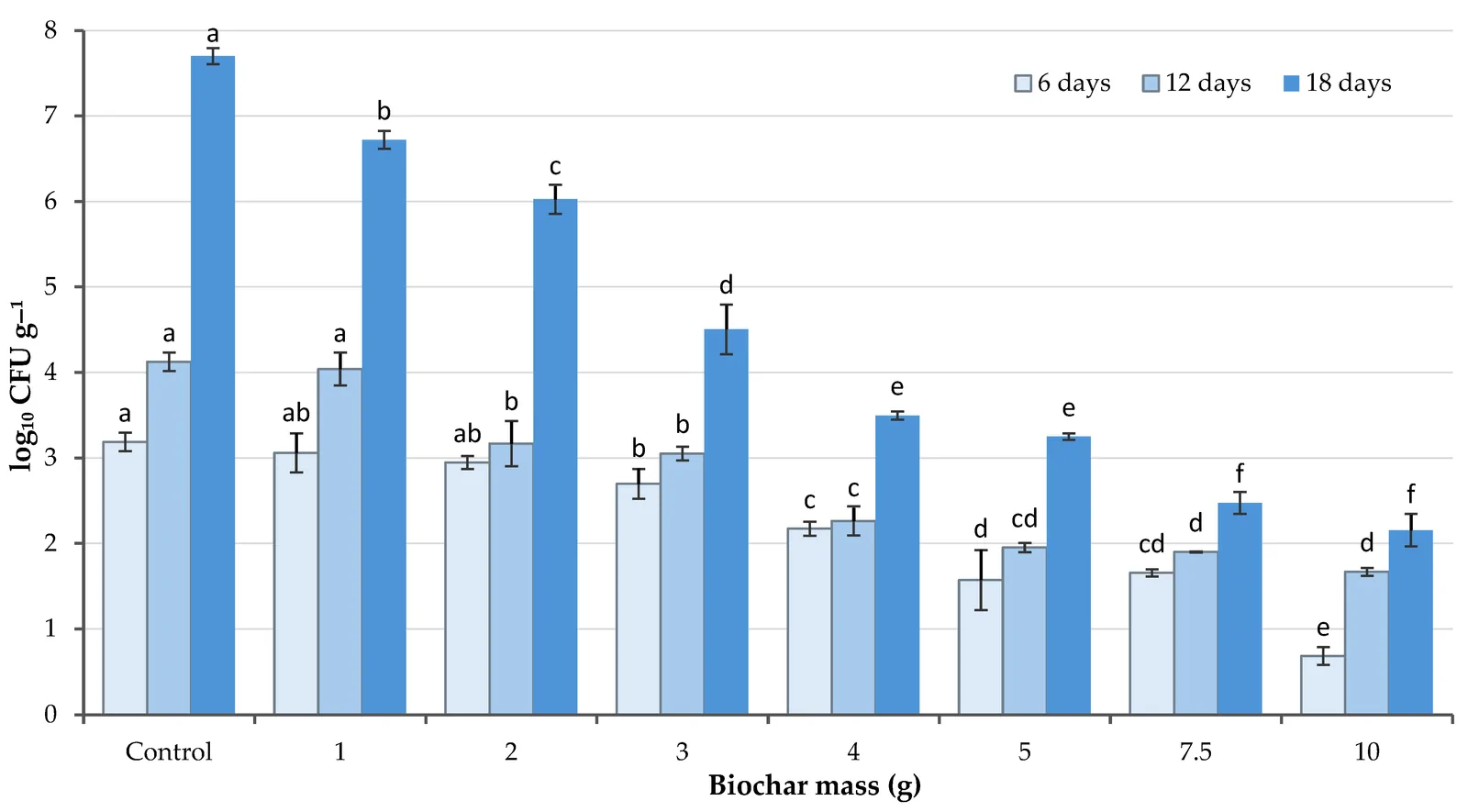
The total count of aerobic mesophilic microorganisms in tomato samples after 6, 12, and 18 days of storage. C—control; 1.0–10.0 g—biochar mass (g). Different lowercase letters indicate statistically significant differences among treatments within the same storage time according to Tukey’s test (*p* < 0.05).

The total count of aerobic mesophilic microorganisms increased with storage time in all treatments (Figure 2). In the control, the count increased from approximately 3.20 log_10_ CFU g^−1^ after 6 days to approximately 7.70 log_10_ CFU g^−1^ after 18 days. Biochar limited this increase, and the magnitude of the effect generally increased with the mass applied. After 6 days, the 1–3 g treatments showed values close to those of the control, whereas a clearer reduction was observed from 4 g onwards. The strongest effect was consistently observed for 7.5 and 10 g of biochar. At the end of storage, microbial counts in these treatments remained below approximately 3.00 log_10_ CFU g^−1^, whereas the control exceeded 7.00 log_10_ CFU g^−1^. Differences among treatments became more pronounced with storage time, and after 18 days all biochar treatments showed significantly lower counts than the control (*p* < 0.05).

**Figure 3:**
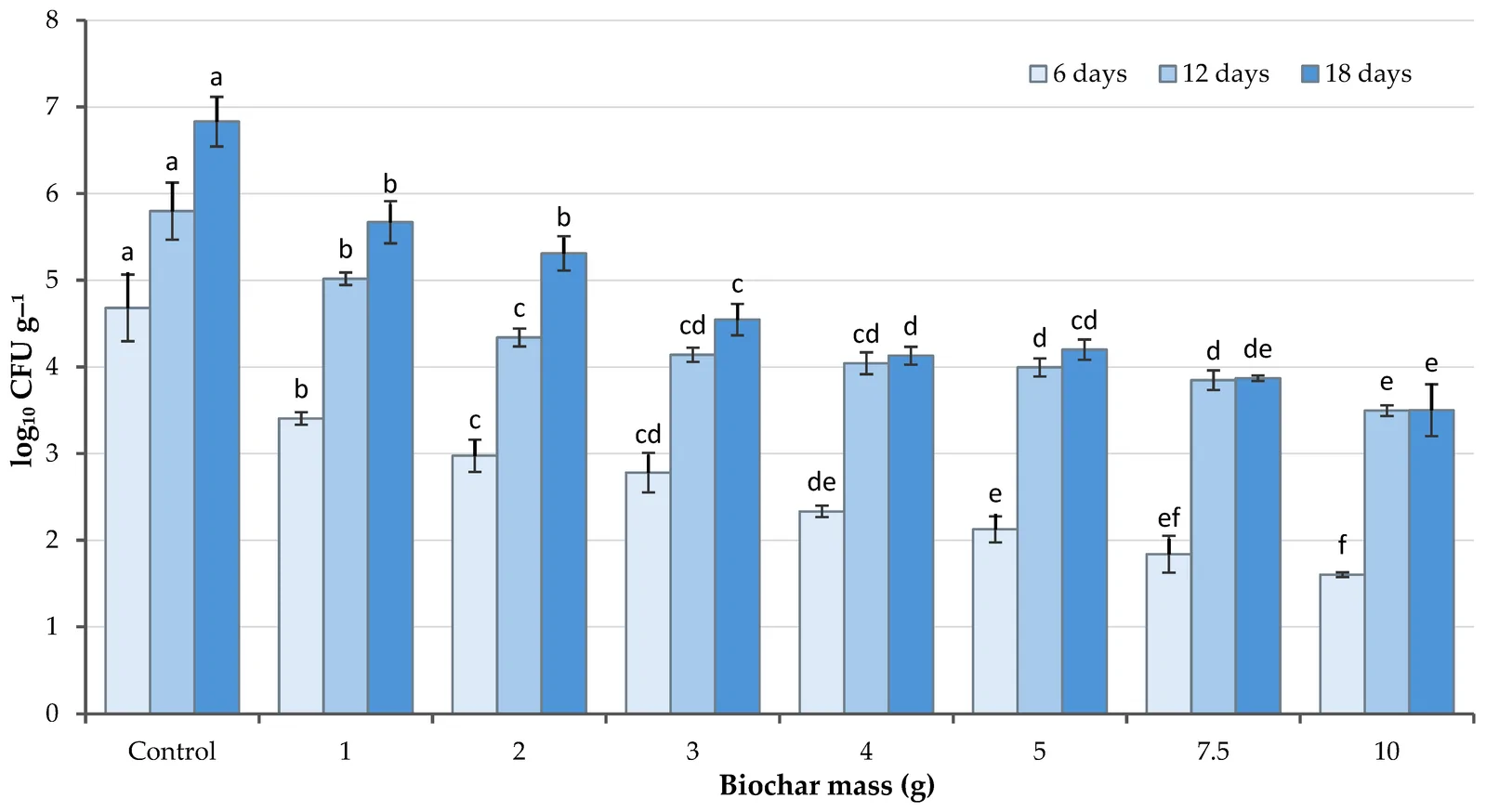
Yeast and mold counts in tomato samples after 6, 12, and 18 days of storage. C—control; 1.0–10.0 g—biochar mass (g). Different lowercase letters indicate statistically significant differences among treatments within the same storage time according to Tukey’s test (*p* < 0.05).

A similar pattern was observed for yeasts and molds (Figure 3). The control showed an increase from approximately 4.70 log_10_ CFU g^−1^ after 6 days to approximately 6.80 log_10_ CFU g^−1^ after 18 days. Biochar-treated samples generally showed lower fungal counts, with the greatest reduction again observed for the 7.5 and 10 g treatments. After 6 days, the 10 g treatment showed approximately 1.60 log_10_ CFU g^−1^ compared with 4.70 log_10_ CFU g^−1^ in the control. Although yeast and mold counts increased during continued storage, the treatments containing the highest biochar masses maintained markedly lower values than the control. Overall, the results indicate a clear mass-dependent tendency, with 7.5 and 10 g providing the greatest limitation of microbial proliferation during prolonged storage (*p* < 0.05).

**Figure 4:**
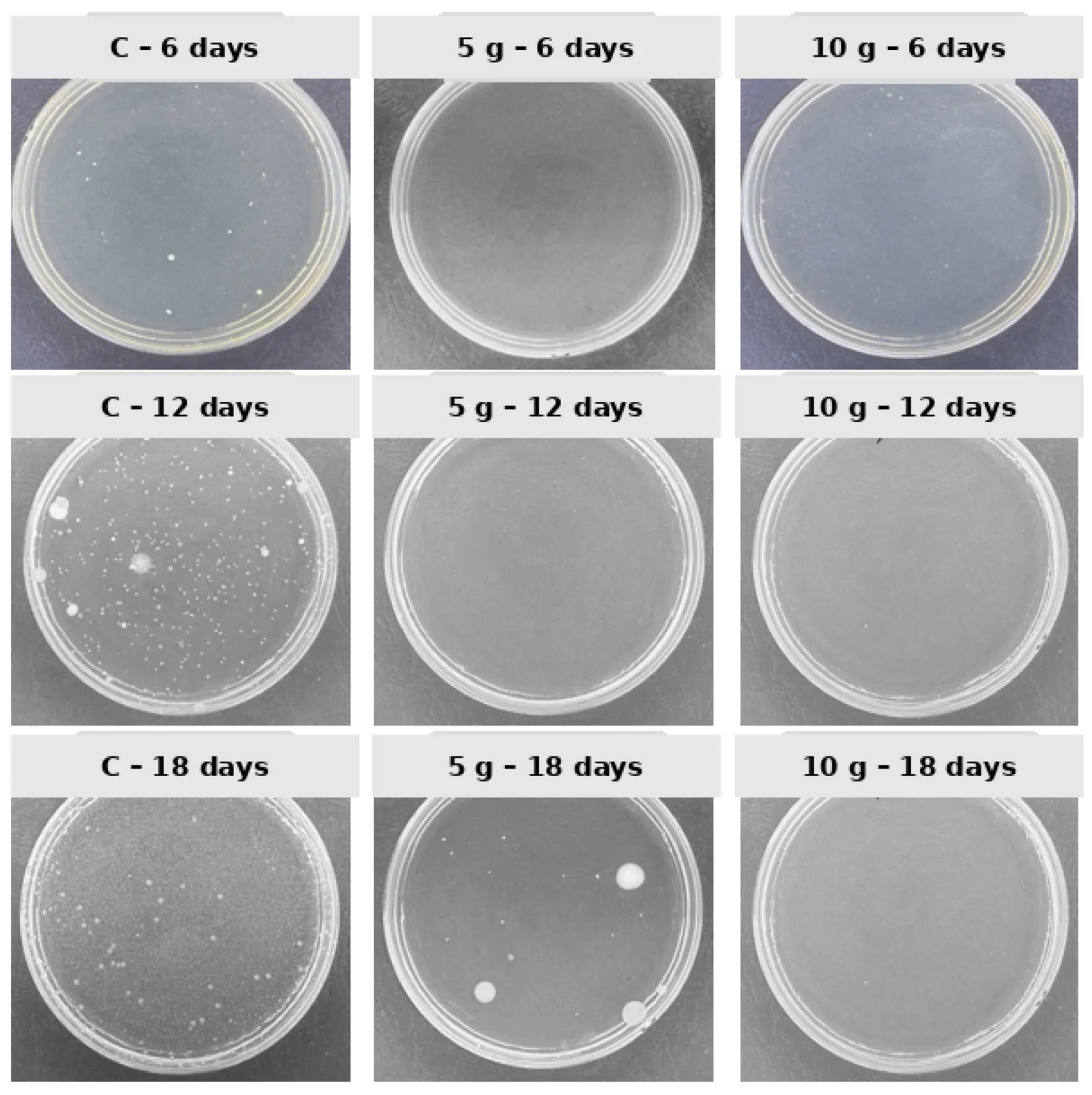
Representative plates used for the enumeration of aerobic mesophilic microorganisms after different storage periods. C—control; 5 g and 10 g—samples stored with the indicated mass of tea-waste biochar. Dilution factor: 10^−1^. Source: authors’ original photograph.

Representative plate images support the quantitative results (Figure 4). Colony development increased with storage time in the control, whereas visibly fewer colonies were present in samples stored with 5 and 10 g of biochar. The effect was particularly pronounced for the 10 g treatment. These observations should be interpreted as evidence of reduced microbial proliferation under the applied storage conditions, rather than complete inhibition, because viable microorganisms were still detected in the quantitative enumeration.

**Figure 5:**
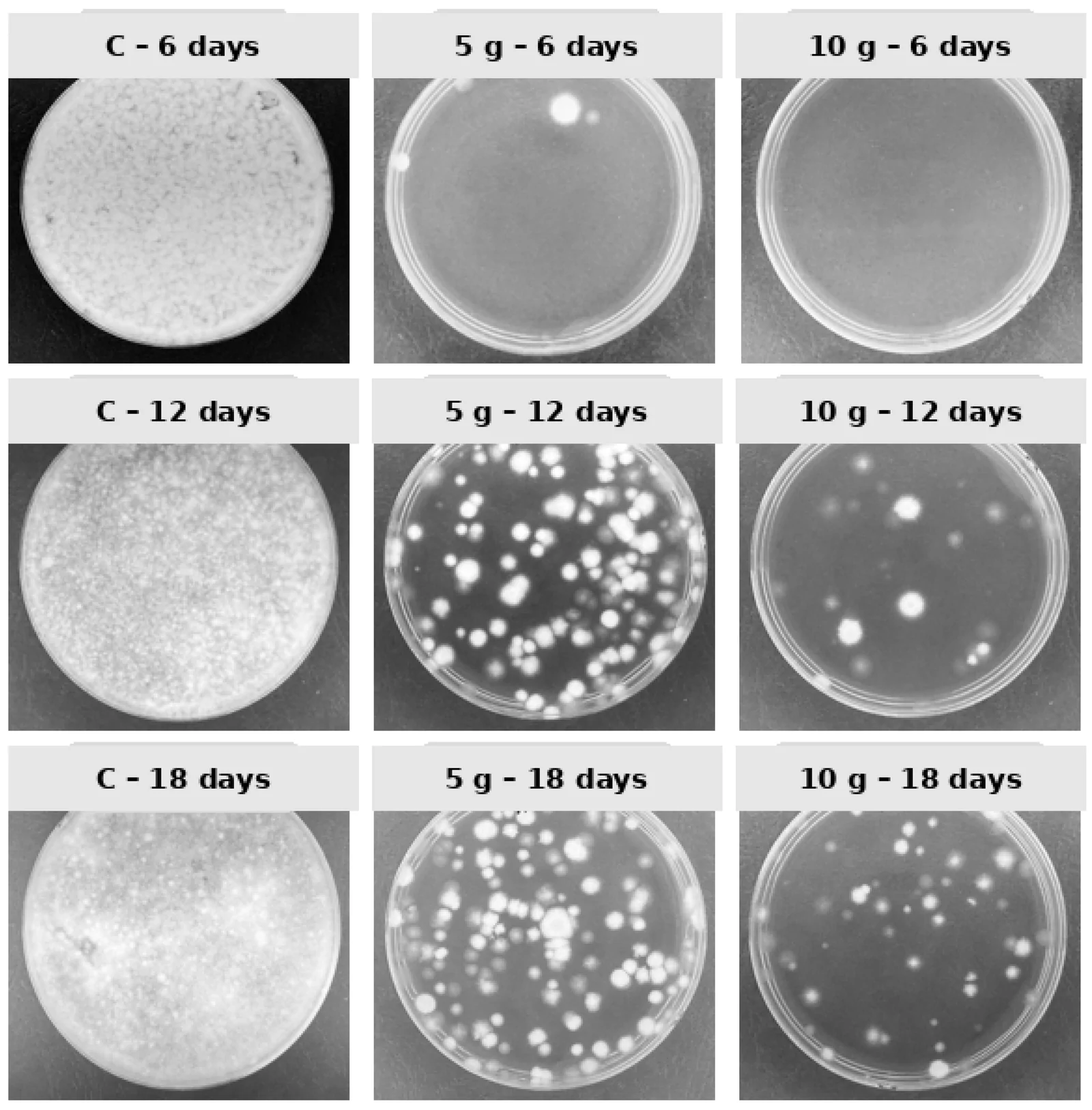
Representative plates used for the enumeration of yeasts and molds after different storage periods. C—control; 5 g and 10 g—samples stored with the indicated mass of tea-waste biochar. Dilution factor: 10^−1^. Source: authors’ original photograph.

The plate images obtained on Sabouraud agar with chloramphenicol further illustrate the reduction in yeast and mold development associated with biochar (Figure 5). After 6 days, no clearly visible fungal colonies were observed on the representative plate from the 10 g treatment, while extensive growth was present in the control. After 12 and 18 days, fungal colonies appeared in all treatments, but their abundance remained lower in the samples stored with 5 and 10 g of biochar than in the control. The progressive increase in fungal counts over time was therefore not completely prevented, but it was clearly attenuated by the higher biochar masses.

Taken together, the microbiological results indicate that tea-waste biochar was associated with a dose-dependent limitation of both bacterial and fungal proliferation during tomato storage. The strongest effect was consistently observed for 7.5 and 10 g of biochar. This pattern may be linked indirectly to the lower ethylene accumulation observed in these treatments, because slower ripening and delayed tissue softening can reduce the availability of nutrients and damaged tissue surfaces that facilitate microbial colonization. The increase in microbial counts observed in the control during prolonged storage is consistent with the general microbial deterioration of ripe tomatoes reported in the literature. Zoellner et al. described mesophilic aerobic bacterial populations on fresh tomatoes within the supply chain, demonstrating that tomatoes may carry substantial and variable background microbiota [44]. Poosarla et al. likewise observed a progressive increase in mesophilic bacteria and fungi during tomato storage, while the application of an active coating limited microbial proliferation and extended shelf life [45]. More recently, Aguerri et al. showed that active paper containing natural antifungal agents significantly reduced mold development and fruit decay during tomato storage [46]. These observations are complemented by studies of carbonaceous materials obtained from agricultural and food-industry waste and used to limit ethylene accumulation and delay fruit ripening. Tepamatr demonstrated that activated carbon derived from sugarcane bagasse effectively removed ethylene, whereas modification with 1% palladium further increased the efficiency of this process. After 10 days of storage, the fruit retained a greener color than in the other treatments [47]. Regadera-Macías et al. showed that activated carbons obtained from olive stones, particularly those activated with CO_2_, possessed favorable properties for ethylene adsorption. The adsorption process was fully reversible, allowing the spent materials to be readily regenerated and reused in filters intended for the agri-food industry [48]. Sriprom et al. applied 10 g of activated carbon derived from mango seeds per 1 kg of fruit. This was the most effective treatment, delaying mango ripening by approximately 5 days compared with the control and maintaining fruit quality for up to 14 days of storage [49]. Although these preservation systems differ from tea-waste biochar, their results support the present finding that materials capable of modifying the storage microenvironment can slow microbial deterioration. The marked reduction observed with the highest biochar masses suggests that this waste-derived material may contribute to maintaining the microbiological quality of tomatoes; however, validation under commercial storage conditions is still required.

### 2.3. Firmness

Tomato firmness decreased during storage, but the extent of this decline depended on the mass of biochar applied. The initial mean firmness was 1.87 kgf. The treatments containing 5, 7.5, and 10 g of biochar showed the highest firmness throughout storage, reaching 1.90, 1.90, and 1.91 kgf after 6 days and 1.77, 1.77, and 1.80 kgf after 18 days, respectively. These results indicate that higher biochar masses most effectively limited firmness loss.

Statistical analysis revealed significant differences among treatments at the individual storage times. After 6 days, the treatments containing 5, 7.5, and 10 g of biochar showed significantly greater firmness than the control. After 12 days, the firmness of the control was significantly lower than that of all biochar treatments. After 18 days, the highest firmness values were recorded in the 5, 7.5, and 10 g treatments; these treatments did not differ significantly from one another, whereas their firmness was significantly greater than that of the control.

**Figure 6:**
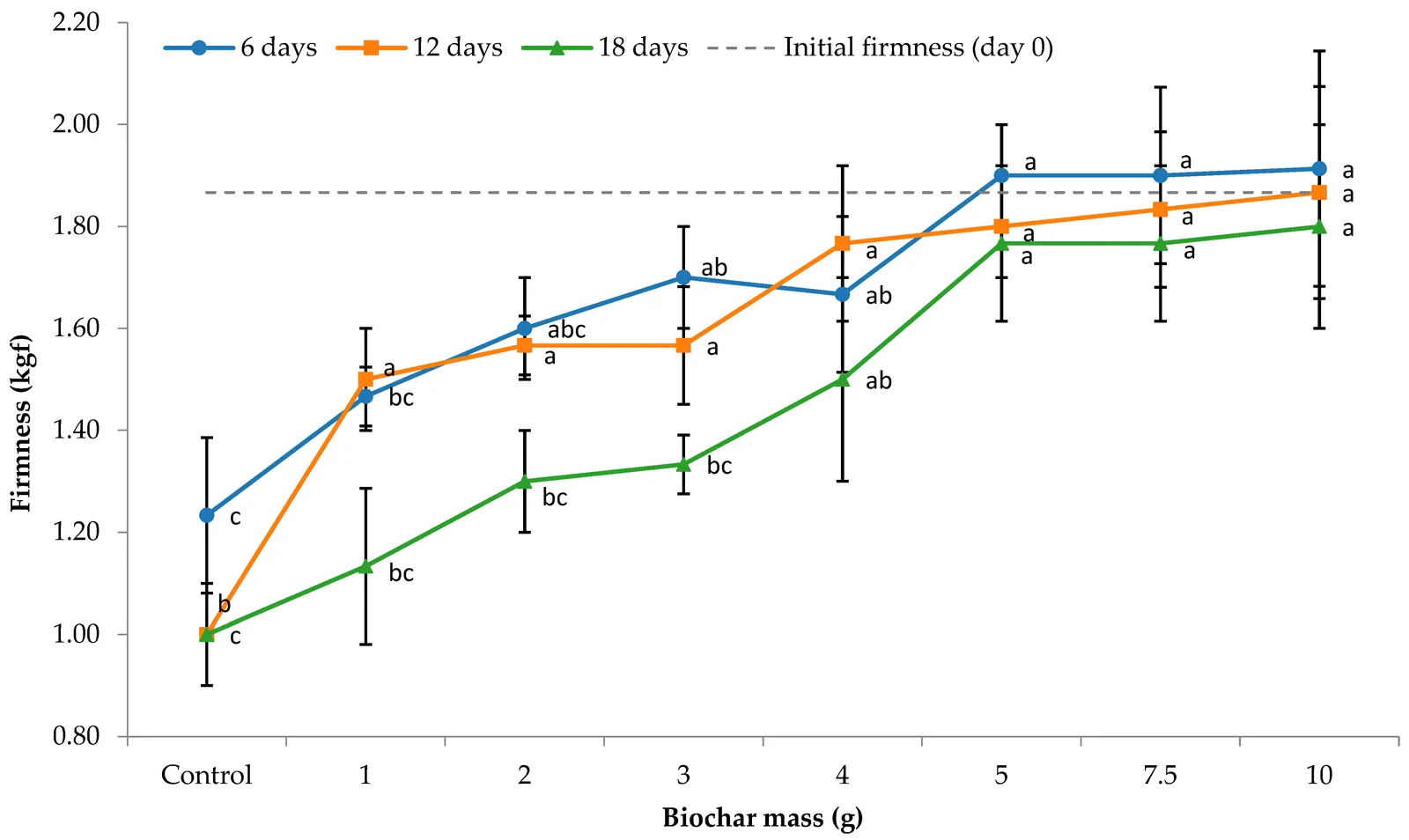
Effect of different amounts of tea-waste biochar on tomato firmness after 6, 12, and 18 days of storage. The horizontal dashed line indicates the initial firmness of tomatoes before storage; this value was presented for reference only and was not included in the statistical analysis. Different lowercase letters indicate statistically significant differences among treatments within the same storage time according to Tukey’s test (*p* < 0.05).

The most rapid firmness loss occurred in the control, where the mean value decreased from the initial 1.87 kgf to 1.23 kgf after 6 days and 1.00 kgf after 12 and 18 days, corresponding to a final decrease of approximately 46.4%. Treatments containing lower bio-char masses showed intermediate firmness losses, whereas the 5, 7.5, and 10 g treatments maintained values close to the initial firmness. The tomato firmness results are presented in Figure 6.

The results indicate that spent-tea-leaf biochar may limit the loss of tomato firmness during storage and that, under the conditions of this experiment, the effectiveness of this effect depended on the amount of material applied. The most likely mechanism is the slowing of processes associated with ripening and water loss, which lead to cell-wall degradation and reduced tissue firmness. The observed relationships are consistent with the scientific literature. Lana et al. (2005) [50] showed that the decline in tomato firmness during storage is associated, among other factors, with increased metabolic activity and the activity of enzymes that degrade cell-wall components. These processes loosen fruit tissue structure and increase susceptibility to water loss [50]. Moisture loss also contributes to fruit wilting, shrinkage, and reduced firmness [51]. Consequently, greater moisture loss is generally associated with a lower capacity to retain firmness, which is consistent with the trends observed in the present experiment. Tigist et al. (2013) [52] further demonstrated that flesh firmness may differ significantly among cultivars and that changes in this parameter are associated with transformations of cell-wall components, particularly pectic substances. Firmness is an important criterion of the commercial quality of tomatoes because it is associated with both consumer acceptance and fruit storability [52].

### 2.4. pH and Titratable Acidity

**Figure 7:**
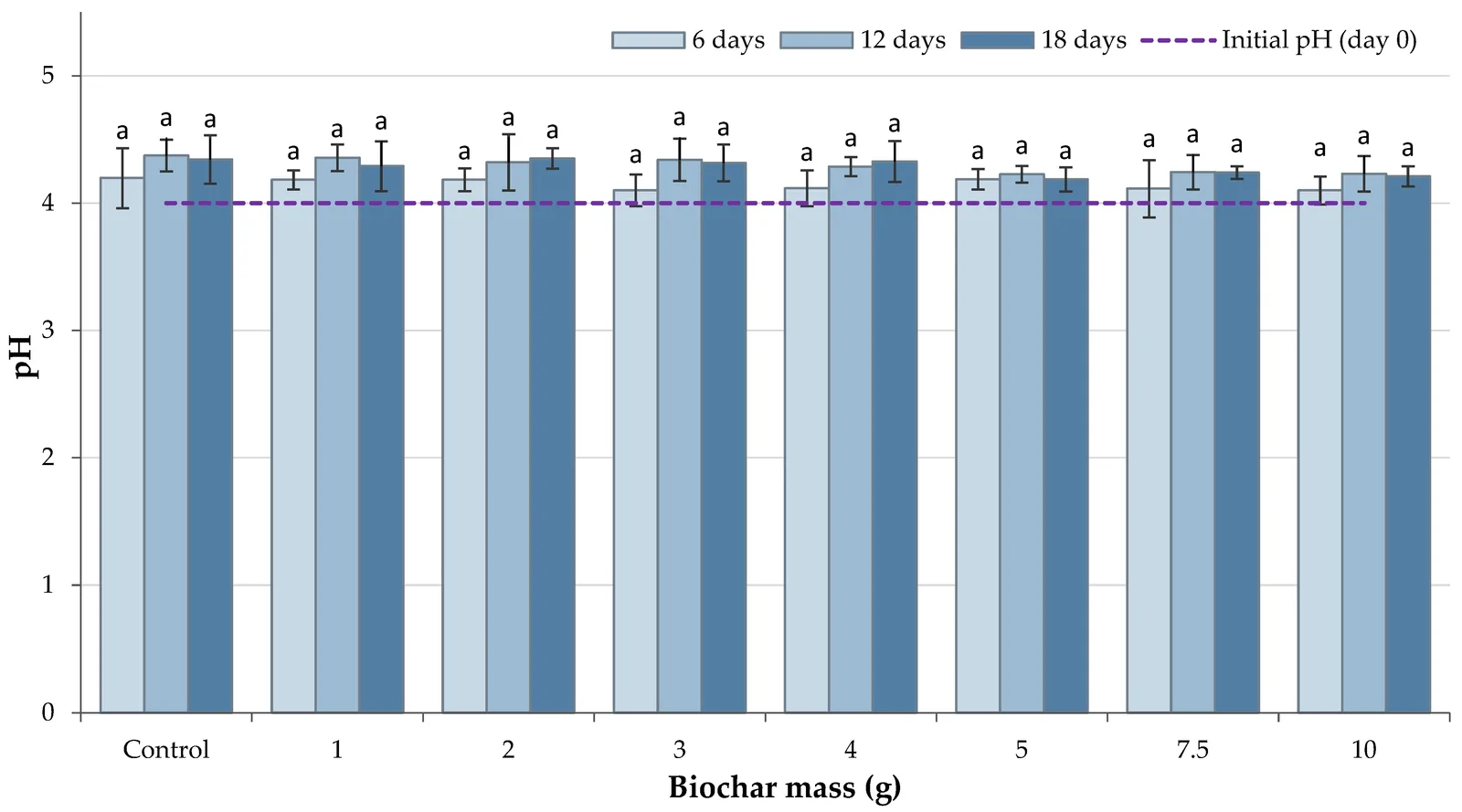
Effect of different amounts of tea-waste biochar on tomato pH after 6, 12, and 18 days of storage. The horizontal dashed line indicates the initial pH of tomatoes before storage; this value was presented for reference only and was not included in the statistical analysis. Different lowercase letters indicate statistically significant differences among treatments within the same storage time according to Tukey’s test (*p* < 0.05).

In fruits and vegetables, pH and titratable acidity are important parameters affecting flavor, sensory quality, and storability [53,54,55]. Tomato acidity results primarily from the presence of organic acids, particularly citric and malic acids [56]. The mean pH of the tomatoes before storage was 4.00 and increased slightly during the experiment. Across all storage times and treatments, pH values remained within a relatively narrow range of 4.10–4.37. Although small differences in mean values were observed among the treatments, no statistically significant differences were found at any storage time (*p* > 0.05). Thus, biochar application had no significant effect on tomato pH, which remained relatively stable throughout storage (Figure 7).

The slight increase in pH observed during storage is consistent with the literature reports. Mohammed et al. (1999) showed that the pH of ripe tomatoes depends on the cultivar and may exceed 4.60 [51]. Haider et al. (2020) reported pH values ranging from approximately 4.00 to 5.30 during tomato storage [26]. Changes in pH during fruit ripening and storage may be associated with transformations of organic acids used as substrates in respiratory processes. The literature indicates that ethylene scavengers may slow fruit ripening and metabolic transformations, which may affect the rates of change in pH and titratable acidity [53,54,55,56,57,58,59]. In the present experiment, however, the applied biochar amounts did not produce a consistent or statistically significant reduction in pH relative to the control. The relatively small changes in pH may have resulted from transformations of organic acids during tomato ripening and storage and from their participation in metabolic and respiratory processes [60,61]. The results therefore suggest that the effect of biochar on tomato quality preservation was more evident for other parameters than for pH, which remained relatively stable throughout the storage period.

**Figure 8:**
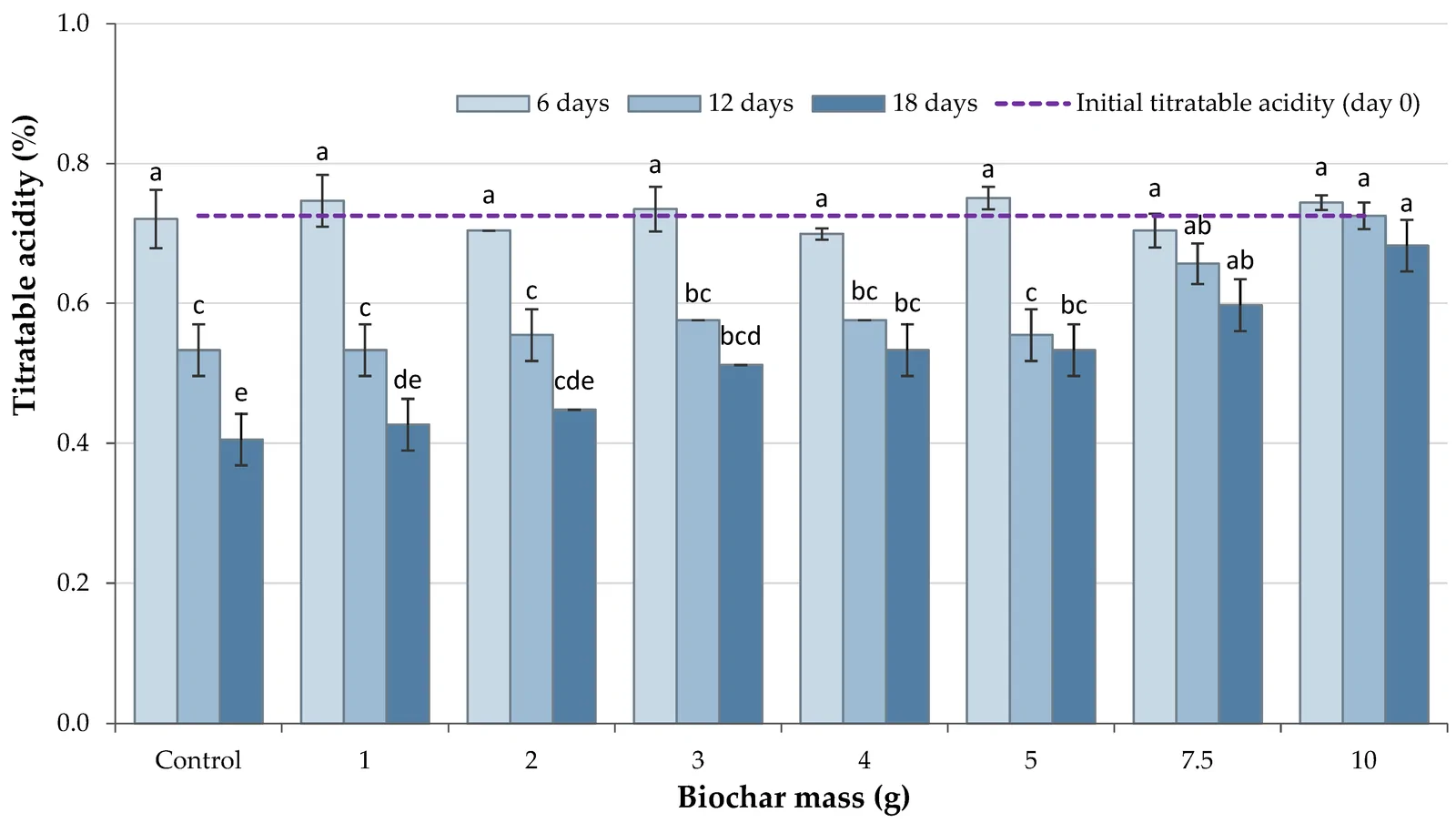
Effect of different amounts of tea-waste biochar on tomato titratable acidity after 6, 12, and 18 days of storage. The horizontal dashed line indicates the initial titratable acidity of tomatoes before storage; this value was presented for reference only and was not included in the statistical analysis. Different lowercase letters indicate statistically significant differences among treatments within the same storage time according to Tukey’s test (*p* < 0.05).

Total soluble solids and titratable acidity are among the principal parameters affecting sweetness, sourness, flavor, and the overall sensory quality of fruit [53]. During tomato ripening and storage, organic acid content generally decreases because these compounds are used as substrates in metabolic and respiratory processes. This phenomenon may be accompanied by a decrease in titratable acidity and an increase in pH [53]. Before storage, the mean titratable acidity of the tomatoes was 0.73%. No significant differences among treatments were observed after 6 days. At later storage times, however, higher biochar masses promoted better retention of titratable acidity. After 12 days, the 7.5 and 10 g treatments showed significantly higher values than the control, whereas after 18 days the treatments containing 3–10 g of biochar were significantly higher than the control. The strongest effect was observed for the 10 g treatment, which retained a titratable acidity of 0.68% after 18 days, approximately 68% higher than the control (0.41%). Changes in titratable acidity are presented in Figure 8.

The results indicate an overall decrease in titratable acidity with increasing storage time. However, this decrease was clearly greater in the control than in treatments containing larger amounts of biochar. The effect of biochar was not yet evident after 6 days, but became statistically significant after 12 days in the 7.5 and 10 g treatments and after 18 days in the treatments containing 3–10 g of biochar. The results therefore suggest that biochar, particularly when applied at higher amounts, contributed to the longer retention of tomato acidity during storage. The slower decline in titratable acidity in the biochar treatments may have been associated with ethylene scavenging and a reduced rate of fruit ripening. Reduced ethylene availability may have slowed metabolic activity and respiration, thereby limiting the use of organic acids as respiratory substrates [61]. These results are consistent with studies on various substances used to reduce ethylene action. Sammi and Masud [57] reported slower weight loss and acidity decline in tomatoes stored with calcium chloride and potassium permanganate. Mujtaba et al. [58] showed that the application of 2% calcium chloride and 800 ppm boric acid promoted the retention of pH and titratable acidity in stored tomatoes. Muhammad et al. [59] investigated the combined use of potassium permanganate and hypobaric conditions during 21 days of tomato storage and obtained improved quality retention, which was associated, among other factors, with the maintenance of appropriate acidity. Melin et al. [62] analyzed the effects of copper- and zinc-based absorbents used in combination with neutral zeolite on the postharvest quality of tomatoes. The authors indicated that increased respiration promotes the degradation of organic acids and changes in soluble solids content. Ethylene-accelerated ripening may therefore shorten the postharvest shelf life of fruit. The increase in pH and simultaneous decrease in titratable acidity observed during storage were associated, among other factors, with a reduction in citric acid content [62].

### 2.5. Total Soluble Solids and Dry Matter

**Figure 9:**
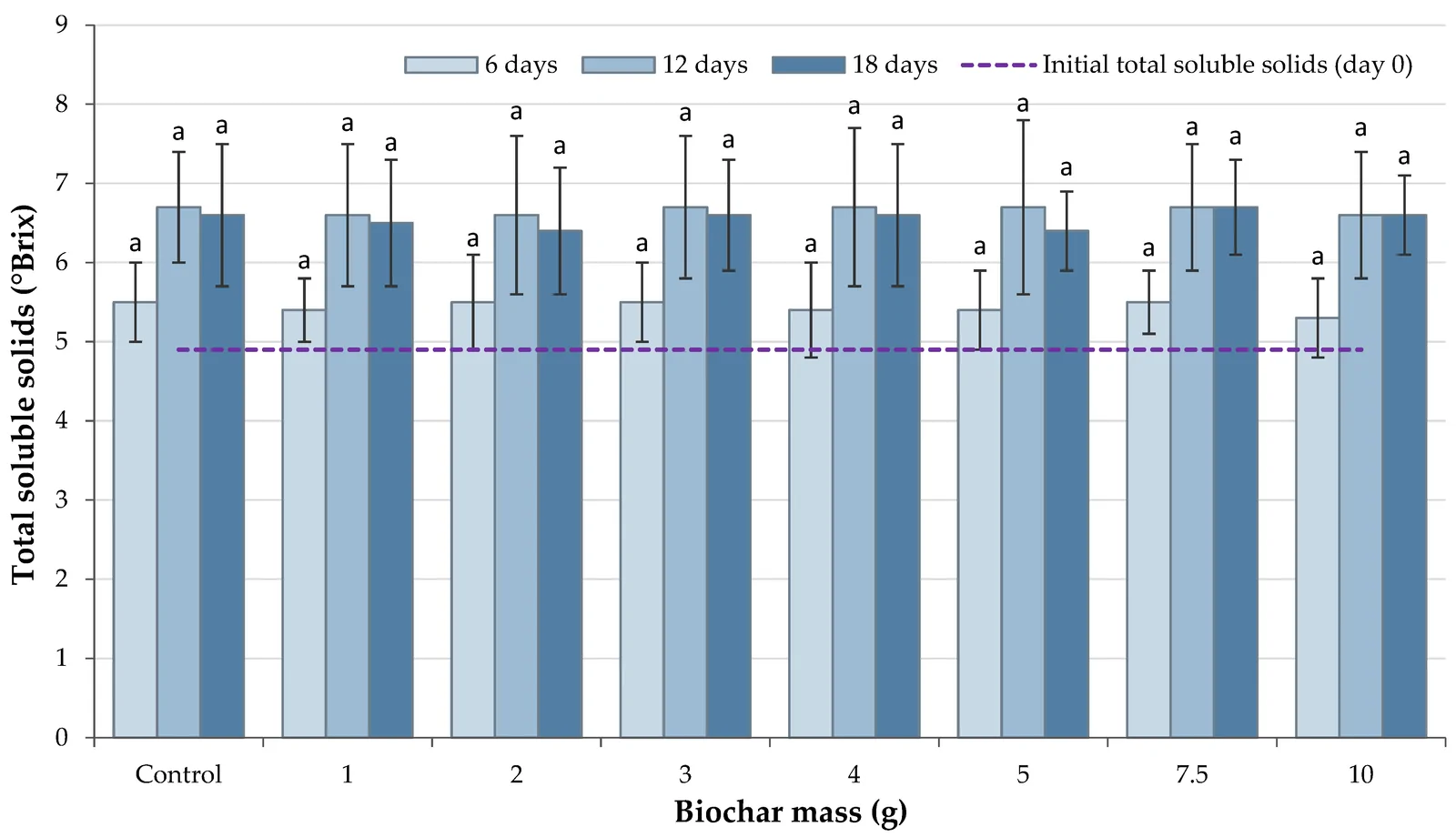
Effect of different amounts of tea-waste biochar on tomato total soluble solids after 6, 12, and 18 days of storage. The horizontal dashed line indicates the initial total soluble solids of tomatoes before storage; this value was presented for reference only and was not included in the statistical analysis. Different lowercase letters indicate statistically significant differences among treatments within the same storage time according to Tukey’s test (*p* < 0.05).

Changes in the total soluble solids content of tomatoes during storage are presented in Figure 9. The initial mean TSS value was 4.90 °Brix. Total soluble solids increased during storage, reaching approximately 6.40–6.70 °Brix by day 18, with the greatest increase occurring between days 6 and 12. No significant differences were found between the control and biochar treatments at any storage time (*p* > 0.05), indicating that biochar mass had no clear effect on tomato TSS.

Sugars are important components of tomatoes because they determine sweetness and contribute substantially to overall flavor. Tomatoes contain primarily glucose and fructose, whereas sucrose is generally present in small amounts. Soluble components of tomato juice also include organic acids, amino acids, and soluble pectins. As ripening progresses and storage time increases, the consumption of sugars and organic acids rises because these compounds are used as substrates in respiratory processes [63]. The increase in TSS during storage is attributed to the hydrolysis of complex carbohydrates into simple sugars, transformations of cellular components, and the concentration of soluble substances resulting from water loss. TSS generally increases during fruit ripening; after reaching a maximum, it may stabilize or gradually decrease [52,64]. The increase in TSS during storage observed in the present experiment is consistent with patterns described in the literature. Zakriya et al. showed that the total soluble solids content of tomatoes changed with increasing storage time, including in treatments in which ethylene scavengers were used [53]. Wills and Ku demonstrated that the application of 1-MCP, which limits ethylene action, did not significantly affect tomato soluble solids content compared with the control [65]. This result is similar to that of the present experiment, in which no significant differences in TSS were found between the control and adsorbent treatments despite biochar application and reduced ethylene concentration. This indicates that changes in TSS were primarily associated with ripening and storage time, whereas the effect of the amount of biochar applied was limited.

**Figure 10:**
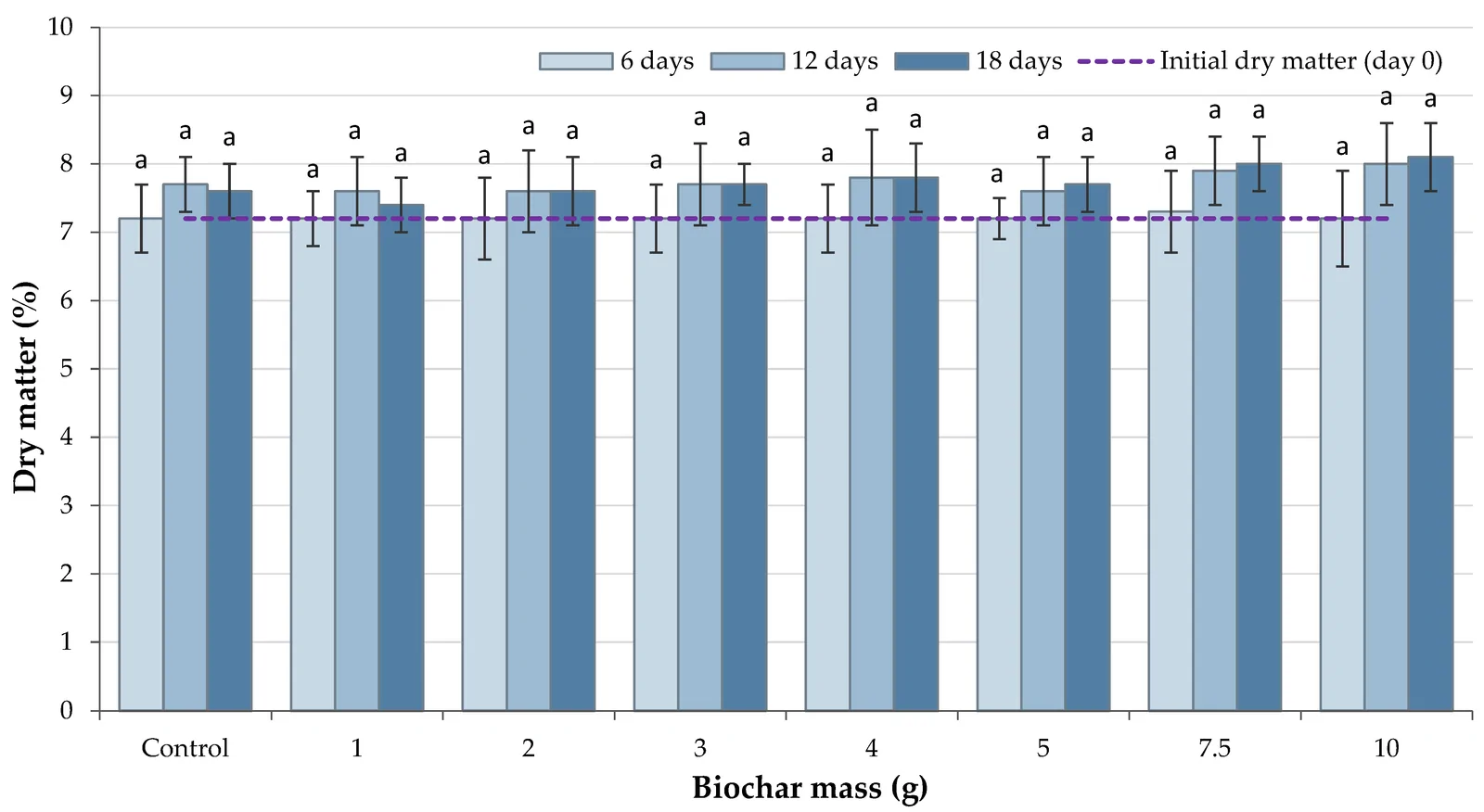
Effect of different amounts of tea-waste biochar on tomato dry matter after 6, 12, and 18 days of storage. The horizontal dashed line indicates the initial dry matter of tomatoes before storage; this value was presented for reference only and was not included in the statistical analysis. Different lowercase letters indicate statistically significant differences among treatments within the same storage time according to Tukey’s test (*p* < 0.05).

The initial dry matter content was 7.20% and increased slightly during storage, reaching approximately 7.40–8.10% after 18 days. Although higher mean values tended to occur in treatments containing greater amounts of biochar, no statistically significant differences between the control and biochar treatments were detected at any storage time (*p* > 0.05). Changes in tomato dry matter content during storage are presented in Figure 10.

Tomato dry matter content may remain relatively stable or increase slightly during storage, depending on the balance between water loss and the consumption of organic compounds in respiratory processes. Toor and Savage showed that the dry matter content of tomatoes stored for 10 days at 7, 15, and 25 °C ranged from 4.8% to 5.4% and did not change significantly during storage [66]. Sinha et al. reported an increase in the percentage of dry matter with increasing storage time, which they attributed to a gradual reduction in fruit water content. The authors demonstrated that the extent of these changes depended on both tomato cultivar and storage method, confirming that dry matter content is strongly associated with fruit water balance during storage [67].

### 2.6. Total Phenolic Content and Antioxidant Activity

Total phenolic content and antioxidant activity were determined before storage and after 6, 12, and 18 days, with the initial values serving as reference values. The initial TPC was 42.10 ± 0.63 mg GAE 100 g^−1^ FW. No significant differences among treatments were observed after 6 days, whereas after 12 days significantly higher TPC values were recorded in the 5, 7.5, and 10 g treatments. This tendency became more pronounced after 18 days, when the highest TPC was observed in the 10 g treatment (53.46 ± 1.71 mg GAE 100 g^−1^ FW), while the lowest values occurred in the control and treatments containing 1–3 g of biochar.

The initial ABTS antioxidant activity was 535.90 ± 13.08 µmol TE 100 g^−1^ FW. Although ABTS activity decreased during storage, the decline was markedly smaller in treatments containing higher biochar masses. After 12 and 18 days, the 7.5 and 10 g treatments showed significantly greater ABTS activity than the control and most lower-dose treatments. At the end of storage, the highest values were recorded for 7.5 and 10 g of biochar, at 484.72 ± 11.83 and 492.50 ± 12.02 µmol TE 100 g^−1^ FW, respectively, compared with 330.19 ± 8.06 µmol TE 100 g^−1^ FW in the control.

The initial DPPH radical-scavenging capacity was 85.56 ± 0.06%. In contrast to the clearer dose-related trends observed for TPC and ABTS, DPPH activity showed greater variability among treatments during storage. After 12 days, the highest value was recorded in the 10 g treatment, whereas after 18 days the highest activity was observed in the 5 g treatment (82.02 ± 1.24%). At the end of storage, relatively high DPPH activity was also maintained in the 4 and 10 g treatments, while the control reached 61.85 ± 1.95%.

The initial FRAP value was 81.07 ± 0.95 µmol TE 100 g^−1^ FW. After 6 days, differences among treatments were relatively small, whereas after 12 days the 7.5 and 10 g treatments showed significantly greater FRAP activity than the other treatments. These treatments also maintained the highest reducing capacity after 18 days, indicating better preservation of antioxidant potential at the highest biochar masses.

The results indicate that the higher biochar amounts, namely 7.5 and 10 g, were associated with better retention of total phenolic content and antioxidant activity during prolonged tomato storage. Detailed results for total phenolic content and antioxidant activity are presented in Table 1.

The higher phenolic content and greater antioxidant activity measured using the ABTS and FRAP assays in the treatments containing 7.5 and 10 g of biochar indicate that greater amounts of this material promoted the retention of tomato antioxidant potential during storage. A similar direction of change has been observed in studies of active ethylene scavengers. Bailén et al. showed that granular activated carbon impregnated with palladium and used in modified-atmosphere packaging reduced ethylene accumulation, slowed color change, softening, and weight loss, and delayed the development of tomato spoilage [42]. Zakriya et al. reported that, after 40 days of storage, tomatoes treated with 2% CaCl_2_ and stored with a sachet containing KMnO_4_ had a total phenolic content of 41.30 ± 0.58 mg GAE 100 g^−1^, whereas the control value was 17.11 ± 0.09 mg GAE 100 g^−1^. In the control, TPC decreased during storage from 33.42 ± 0.20 to 17.11 ± 0.09 mg GAE 100 g^−1^, whereas TPC increased in all treatments involving ethylene scavengers [53]. These findings are consistent with the better retention of TPC and ABTS and FRAP activity observed in the present study at the highest biochar amounts. Similar relationships were described by Mansourbahmani et al., who evaluated the effects of palladium-modified nanozeolite, KMnO_4_-containing nanozeolite, 1-MCP, CaCl_2_, salicylic acid, and UV-C radiation on tomato quality during 35 days of storage. Among the investigated approaches to limiting ethylene, 5% palladium-modified nanozeolite was the most effective. This treatment favorably affected phenolic content, firmness, and weight loss and delayed lycopene accumulation, indicating slower ripening. The authors further showed that phenolic content and antioxidant activity generally decreased with increasing storage time, whereas the control exhibited the lowest antioxidant activity at successive measurement times [68]. This is consistent with the decline in antioxidant potential observed during storage in the present study and its better retention in tomatoes stored with greater amounts of biochar.

The effect of postharvest treatments on tomato phenolic content and antioxidant activity may depend on the technology applied and the storage duration. Xylia et al. evaluated tomatoes stored for 14 days at 11 °C after treatment with a preparation containing rosemary and eucalyptus essential oils, applied either as vapor or by fruit immersion. The authors found no consistent effect of the treatments on TPC, whereas the response of FRAP and DPPH activity depended on the application method and analysis time. This indicates that technologies that delay quality deterioration do not necessarily affect all indicators of antioxidant potential in the same manner [69]. A direction of change comparable to that observed in the present experiment was reported by Pineda-Hidalgo et al. for ‘Keitt’ mango fruit subjected to hot-water treatment, stored for 20 days at 5 °C, and subsequently held for 7 days at 21 °C. After cold storage and ripening, ABTS activity was 85.90 ± 11.80 µmol TE 100 g^−1^ FW in the control and 106.60 ± 8.80 µmol TE 100 g^−1^ FW after treatment, whereas FRAP values were 38.80 ± 10.30 and 55.10 ± 4.70 µmol TE 100 g^−1^ FW, respectively. These results indicate that an appropriately selected postharvest treatment may promote the retention of fruit antioxidant activity during storage [70].

In the present experiment, the most consistent relationship with biochar amount was found for TPC, ABTS, and FRAP, particularly after 12 and 18 days, whereas the DPPH results were more variable. Overall, however, the results indicate that greater amounts of biochar, particularly 7.5 and 10 g, promoted the retention of antioxidant compounds and properties in tomatoes during prolonged storage. The beneficial effect of biochar on the retention of tomato antioxidant properties may have been partly associated with reduced ethylene accumulation in the storage headspace, which could have slowed fruit ripening and senescence and promoted better retention of compounds with antioxidant activity [71].

### 2.7. Visual Appearance of Tomatoes During Storage

**Figure 11:**
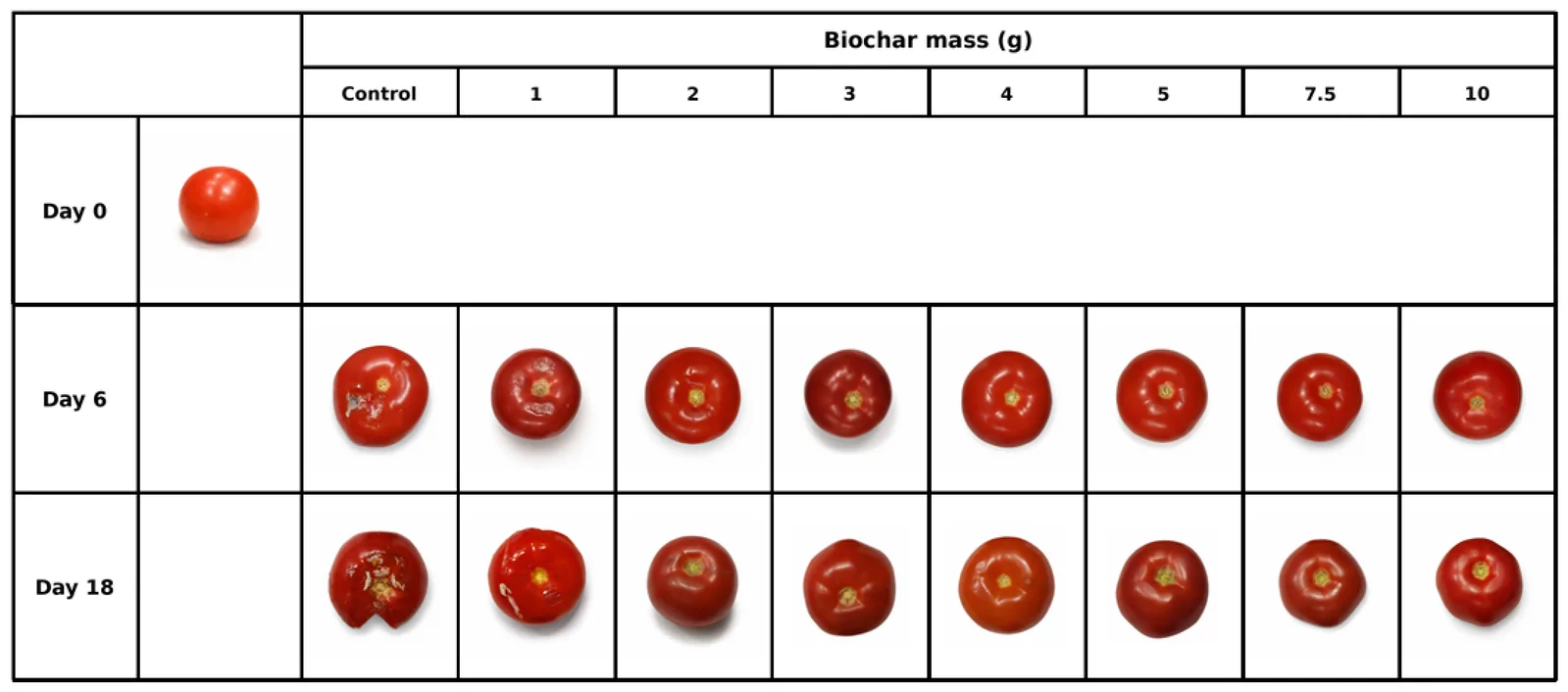
Representative appearance of tomatoes before storage (day 0) and after 6 and 18 days of storage without biochar (control) and with different biochar doses. Source: authors’ original photograph.

Representative photographs of tomatoes taken at selected storage stages are presented in Figure 11. The photographs were intended as qualitative visual documentation and were not used for quantitative color or decay assessment. They complement the instrumental measurements of firmness and the quantitative microbiological analyses presented above. By day 18, the control fruit exhibited the most pronounced deterioration, including extensive surface damage, localized whitish lesions suggestive of microbial spoilage, and partial loss of tissue integrity. In contrast, tomatoes stored with biochar generally retained a more intact and uniform appearance, particularly at the higher biochar doses.

## 3. Materials and Methods

### 3.1. Preparation and Basic Characterization of Tea-Waste Biochar

Biochar was produced from spent residues remaining after the brewing of loose-leaf black tea. The material was prepared under laboratory conditions, which allowed control over the origin of the feedstock, its preparation, and storage conditions before pyrolysis. The feedstock was air-dried under laboratory conditions to a moisture content of approximately 15%.

The prepared material was pyrolyzed at 400 °C for 10 min, defined as the residence time of the feedstock in the furnace, using an FCF 2R retort furnace (CZYLOK, Jastrzębie-Zdrój, Poland) for thermal treatment. The residence time was selected on the basis of preliminary biochar production trials conducted at 400 °C for 5, 10, and 15 min. The biochar obtained after 10 min showed carbon content and mineral composition comparable to those of the material produced after 15 min, whereas extending the residence time resulted in greater mass loss and higher energy input without a corresponding improvement in these basic characteristics. Therefore, 10 min was selected as the most favorable compromise between the properties of the resulting biochar and the energy and material efficiency of the pyrolysis process. The volume of a single batch was approximately 0.50 dm^3^. The process was conducted under a continuous flow of 99.99% pure nitrogen at 10 L min^−1^.

The resulting biochar was ground using a mechanical laboratory mill (IKA-Werke GmbH & Co. KG, Staufen, Germany) to obtain particles smaller than 1 mm and then sieved through a 1 mm mesh. The material was rinsed several times with demineralized water to remove possible surface contaminants and dried at 75 °C for 12 h.

The prepared biochar was weighed in amounts of 1.0, 2.0, 3.0, 4.0, 5.0, 7.5, and 10.0 g and placed in biodegradable cellulose sachets (Mount Everest Tea Company GmbH, Elmshorn, Germany). In this manner, seven ethylene-scavenger treatments differing in the mass of biochar applied were prepared.

For the basic characterization of the material, total carbon, hydrogen, and nitrogen contents, as well as ash, volatile matter, and selected macro- and microelement contents, were determined. Ash and volatile matter contents were measured by thermogravimetric analysis using a TGA 701 analyzer (LECO Corporation, Saint Joseph, MI, USA). Total carbon, hydrogen, and nitrogen contents were determined using a TrueSpec CHN analyzer (LECO Corporation, Saint Joseph, MI, USA). The analyses were performed in accordance with PN-EN ISO 16948:2015-07 [72], PN-EN ISO 18122:2023-05 [73], and PN-EN ISO 18123:2023 [74].

The elemental composition of the biochar was determined after microwave digestion of the samples. A 0.2 g portion of material was weighed into each digestion vessel, and a blank sample was prepared for each analytical series. Digestion was performed using an Ethos One microwave system (Milestone, Sorisole, Italy) with a temperature program designed for biological samples; the process temperature did not exceed 220 °C. After digestion, the vessels were allowed to cool to room temperature, and the digests were diluted to 50 mL with demineralized water. Element concentrations were determined by inductively coupled plasma optical emission spectrometry (ICP-OES) using a Thermo Scientific iCAP 6500 Duo (Thermo Fisher Scientific, Cambridge, UK) spectrometer equipped with a horizontal plasma configuration and capable of axial and radial measurements. Before each series of 10 samples, the instrument was calibrated using certified standard solutions supplied by Merck. A three-point calibration curve covering the expected concentration range of the analyzed samples was used for each element. Yttrium and ytterbium ions at concentrations of 2 and 5 mg L^−1^, respectively, were used as internal standards. The results were corrected for blank values and expressed as mg 100 g^−1^ dry matter of biochar. The limit of quantification for individual elements was 0.01 mg kg^−1^. The proximate and elemental composition and the contents of selected elements in the biochar are presented in Table 2.

### 3.2. Plant Material

Commercially available, fully ripe, red raspberry-type tomatoes (*Solanum lycopersicum* L.), cultivar Madano, were purchased from a local supermarket in the Podkarpackie Voivodeship, Poland, and used in the study. The fruit originated from a single commercial batch. Fully ripe red-stage tomatoes were selected to reflect a realistic consumer scenario, as fruit at this maturity stage are commonly available in retail markets and may subsequently be stored under household conditions before consumption. Tomatoes with similar color, size, shape, and degree of ripeness were selected for the experiment, and fruit showing visible mechanical damage, skin cracking, or signs of microbial spoilage were excluded.

### 3.3. Experimental Design and Storage Conditions

A total of 25 kg of tomatoes was used in the experiment. One kilogram of fruit was placed in each of 24 cloches, representing eight experimental treatments with three replicates each. Individual fruit dimensions were not recorded before the experiment; however, the tomatoes were selected for comparable size, shape, color, and ripeness, and the fruit assigned to each cloche was weighed to obtain a total mass of 1 kg per experimental unit. An additional 1 kg sample was analyzed immediately before storage as the initial sample. The remaining fruit was distributed among 24 tightly sealed glass vacuum cloches. The cloches were used as airtight storage chambers; no vacuum was generated during the experiment. The actual internal volume of each cloche was 5.6 dm^3^.

**Figure 12:**
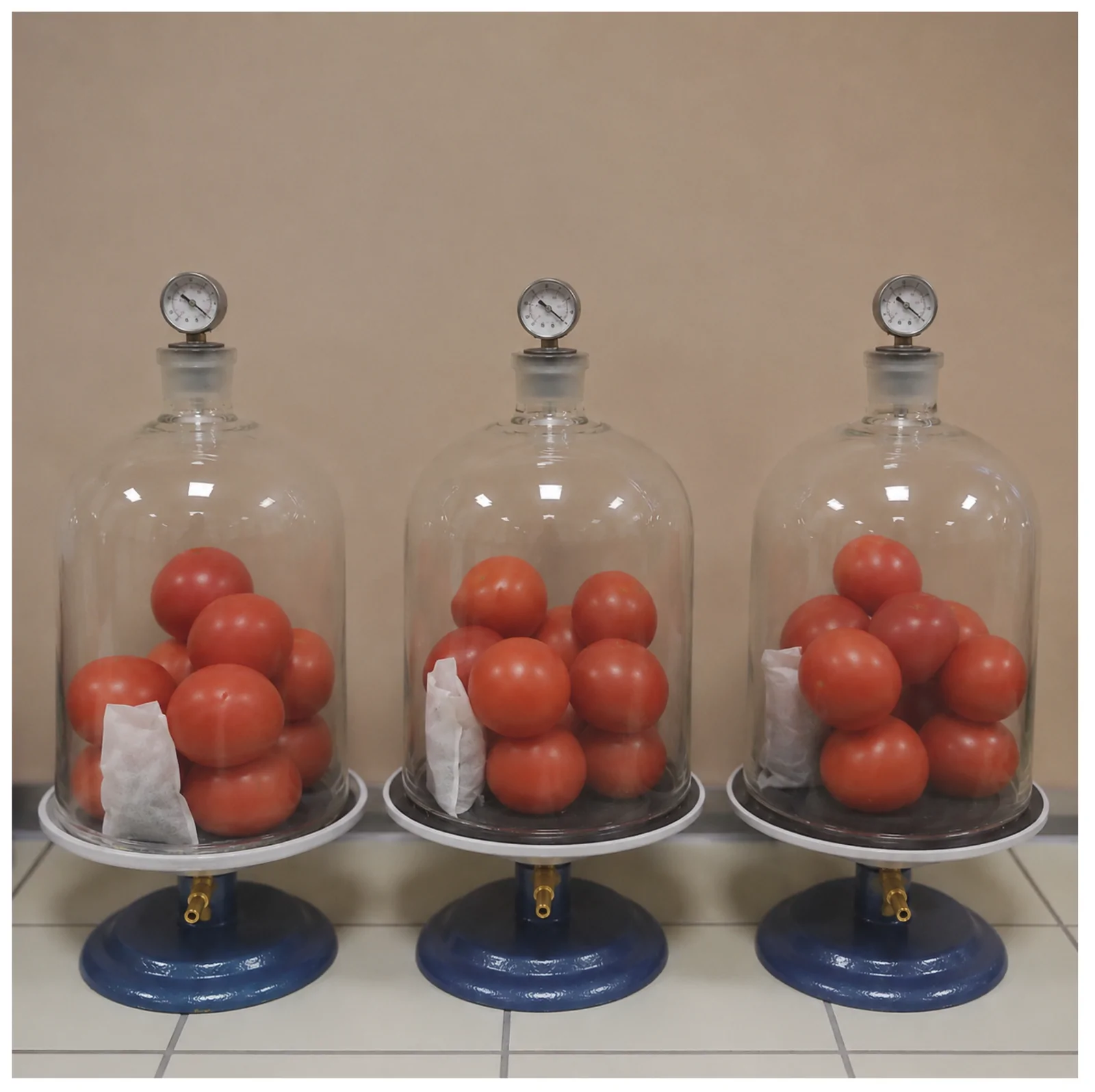
Experimental setup with tomatoes and sachets containing biochar. Source: authors’ original photograph.

The experiment included eight treatments: seven treatments with sachets containing 1.0–10.0 g of biochar and one control treatment without a sachet. The experimental unit was an individual storage cloche, and the three cloches assigned to each treatment constituted three biological replicates (*n* = 3). The tomatoes were stored for 18 days at room temperature (21 °C). Samples were collected after 6, 12, and 18 days of storage from each of the three cloches assigned to a given treatment. At each sampling time, three tomatoes were removed from each cloche for analysis, while the remaining fruit were left in the same chambers until the next sampling time. After each sampling, the cloches were immediately resealed. Photographic documentation of the experimental setup is presented in Figure 12.

### 3.4. Sample Collection and Preparation

At each storage time, tomatoes were collected from each of the three independent cloches assigned to the individual treatments and from the control. Physical attributes were analyzed using whole fruit, whereas appropriately homogenized samples were prepared for chemical, spectrophotometric, and microbiological analyses. The tomatoes were blended using a Zelmer ZSP 4850 blender (B&B Trends S.L., Santa Perpètua de Mogoda, Barcelona, Spain) until a homogeneous sample was obtained, which was then used for further chemical and spectrophotometric analyses.

Representative photographs of the tomatoes were taken at the beginning of the experiment and after selected storage periods under standardized lighting and camera settings. The photographs were used for qualitative documentation of changes in fruit appearance, including color, surface condition, shriveling, and visible signs of deterioration.

### 3.5. Chemicals

2,2′-Azino-bis(3-ethylbenzothiazoline-6-sulfonic acid) (ABTS), 2,2-diphenyl-1-picrylhydrazyl (DPPH), 2,4,6-tris(2-pyridyl)-s-triazine (TPTZ), acetic acid, sodium acetate, hydrochloric acid, potassium persulfate, (±)-6-hydroxy-2,5,7,8-tetramethylchromane-2-carboxylic acid (Trolox), gallic acid, methanol, ethanol, phenolphthalein solution (1%, *w*/*v*), sodium hydroxide (0.1 mol L^−1^), Folin–Ciocalteu reagent, sodium carbonate, iron(III) chloride hexahydrate (FeCl_3_·6H_2_O), sodium chloride, and nitric acid (65%, HNO_3_) were used. All chemicals and solvents were of analytical grade. Plate Count Agar and Sabouraud agar with chloramphenicol were obtained from BioMaxima S.A. (Lublin, Poland). Certified multi-element calibration solutions and yttrium and ytterbium internal-standard solutions used for ICP-OES analysis were obtained from Merck (Darmstadt, Germany). Nitrogen of 99.99% purity was used during pyrolysis, and demineralized water was used for solution preparation and dilution.

### 3.6. Determination of Ethylene Concentration

The ethylene concentration in the atmosphere inside the cloches was measured using a portable DP-35 gas meter equipped with an ethylene (C_2_H_4_) sensor (NANOSENS Sp. z o.o., Tarnowo Podgórne, Poland). A gas sample was drawn through the cloche valve without compromising chamber tightness, and the value was recorded after the instrument reading had stabilized. Measurements were performed after 6, 12, and 18 days of storage, immediately before opening the cloches and collecting fruit for further analyses. The results were expressed in ppm.

### 3.7. Microbiological Analyses

Microbiological analyses were performed after 6, 12, and 18 days of storage. A 20 g tomato sample was aseptically collected from each treatment and replicate, placed in a sterile homogenization bag, and supplemented with 180 mL of sterile 0.85% sodium chloride solution. The samples were homogenized for 210 s using a BagMixer 400 homogenizer (Interscience, Saint-Nom-la-Bretèche, France). Tenfold serial dilutions were prepared from the resulting homogenate in sterile saline solution and subsequently plated on the appropriate microbiological media.

The total count of aerobic mesophilic microorganisms was determined by the pour-plate method on Plate Count Agar (BioMaxima S.A., Lublin, Poland), in accordance with ISO 4833-1:2013/Amd 1:2022 [75]. The plates were incubated aerobically at 30 °C for 72 h, after which the colonies were counted. This standard specifies the enumeration of microorganisms by the pour-plate technique and incubation at 30 °C [75].

Yeasts and molds were enumerated on Sabouraud agar with chloramphenicol (BioMaxima S.A., Lublin, Poland), in accordance with ISO 21527-1:2008. The plates were incubated at 25 °C for 5 days, after which yeast and mold colonies were counted. ISO 21527-1:2008 applies to products with a water activity greater than 0.95, including fresh fruits and vegetables [76]. The results of both analyses were expressed as log_10_ CFU g^−1^ of sample.

### 3.8. Determination of Firmness

Tomato firmness was measured using a handheld penetrometer GY-1 (Wenzhou Sanhe Measuring Instrument Co., Ltd., Wenzhou, China) equipped with an 8 mm diameter probe. Before measurement, a thin section of the peel and adjacent tissue, approximately 2 mm thick, was removed from the equatorial region of each fruit. The probe was then inserted into the tomato flesh to the depth indicated by the mark on the plunger, and the maximum penetration force was recorded. Measurements were performed by the same operator for all samples to minimize operator-related variability. The results were expressed in kgf.

### 3.9. Determination of pH

The pH of the tomato samples was measured using a benchtop pH meter AH 1264 (Adwa Instruments, Szeged, Hungary) according to AOAC Method 981.12 [23]. Before each measurement, the electrode was rinsed with distilled water and gently blotted dry with lint-free tissue. The electrode was then immersed in the homogenized tomato sample, and the pH value was recorded after the reading had stabilized.

### 3.10. Determination of Titratable Acidity

Titratable acidity was determined according to AOAC Method 942.15 [23]. A 1 mL aliquot of the homogenized tomato sample was diluted with 9 mL of distilled water. After the addition of phenolphthalein as an indicator, the sample was titrated with 0.1 mol L^−1^ sodium hydroxide solution until a persistent pale-pink endpoint was reached. The results were converted and expressed as percentages.

### 3.11. Determination of Total Soluble Solids and Dry Matter

Total soluble solids (TSS) and dry matter content were determined nondestructively on intact tomato fruits using an F-750 Produce Quality Meter (Felix Instruments–Applied Food Science, Camas, WA, USA) based on visible and near-infrared spectroscopy. Measurements were performed using the calibration models selected for tomatoes for the determination of TSS and dry matter. Each fruit was placed against the optical window of the instrument, and the values were recorded after completion of the scan. TSS was expressed as °Brix, whereas dry matter content was expressed as a percentage.

### 3.12. Preparation of Extracts for Spectrophotometric Analyses

For each experimental treatment and biological replicate, 5.0 g of homogenized tomato sample was mixed with 20 mL of 96% (*v*/*v*) ethanol in a flask. The mixtures were shaken overnight and subsequently filtered through Whatman No. 1 filter paper (Cytiva, Maidstone, UK). The resulting extracts were used for spectrophotometric analyses.

### 3.13. Determination of Total Phenolics

The total phenolic content (TPC) of the tomato extracts was determined using the Folin–Ciocalteu method, with minor modifications, according to Kaur and Kapoor [77]. A 0.1 mL aliquot of the extract was mixed with 6.0 mL of distilled water, 0.5 mL of Folin–Ciocalteu reagent, and 1.5 mL of saturated sodium carbonate solution. The volume was adjusted to 10 mL with distilled water, and the mixture was incubated in the dark at room temperature for 2 h. Absorbance was measured at 765 nm using a UV–Vis spectrophotometer UV-5100 (Shanghai Metash Instruments Co., Ltd., Shanghai, China). Gallic acid was used as the calibration standard, and the results were expressed as mg GAE 100 g^−1^ FW.

### 3.14. In Vitro Antioxidant Activity

Three complementary assays (ABTS, DPPH, and FRAP) were used to provide a broader assessment of antioxidant activity because they reflect different aspects of antioxidant behavior. ABTS can be applied to evaluate antioxidants with different solubility characteristics, DPPH provides complementary information on the ability of the extracts to react with the DPPH radical in an organic medium, and FRAP evaluates the ferric-reducing power of the extracts, reflecting their electron-donating capacity.

#### 3.14.1. ABTS Radical Scavenging Activity

ABTS radical scavenging activity was determined according to Re et al. [78], with minor modifications. The ABTS radical cation (ABTS•+) was generated by reacting ABTS with potassium persulfate and incubating the mixture in the dark at room temperature for 16 h. Before analysis, the solution was diluted with methanol to an absorbance of 1.000 ± 0.020 at 414 nm. Subsequently, 20 µL of the tomato extract was mixed with 980 µL of the diluted ABTS•+ solution and incubated at room temperature for 6 min. Absorbance was measured at 414 nm using a UV–Vis spectrophotometer (UV-5100, Biosens, Poland). Trolox was used as the calibration standard, and the results were expressed as µmol Trolox equivalents per 100 g of fresh weight (µmol TE 100 g^−1^ FW).

#### 3.14.2. DPPH Radical Scavenging Activity

DPPH radical scavenging activity was determined according to Bartosz [79], with minor modifications. A 0.1 mM DPPH solution was prepared in methanol. Subsequently, 100 µL of the tomato extract was mixed with 900 µL of the DPPH solution, thoroughly mixed, and incubated in the dark at room temperature for 30 min. Absorbance was measured at 517 nm using a UV–Vis spectrophotometer (UV-5100, Biosens, Poland). DPPH inhibition was calculated according to Equation (1):

DPPH inhibition (%) = [(A_0_ − A_1_)/A_0_] × 100 (1)

where A_0_ is the absorbance of the control containing the DPPH solution without tomato extract, and A_1_ is the absorbance of the reaction mixture containing the tomato extract.

The results were expressed as DPPH inhibition (%).

#### 3.14.3. Ferric Reducing Antioxidant Power (FRAP) Assay

Ferric reducing antioxidant power was determined according to Bartosz [79], with minor modifications. The FRAP reagent was prepared immediately before analysis by mixing 300 mM sodium acetate buffer (pH 3.6), 10 mM TPTZ solution, and 20 mM FeCl_3_·6H_2_O solution in a volume ratio of 10:1:1. Subsequently, 40 µL of the tomato extract was mixed with 1160 µL of the freshly prepared FRAP reagent, thoroughly mixed, and incubated in the dark at room temperature for 20 min. Absorbance was measured at 593 nm using a UV–Vis spectrophotometer (UV-5100, Biosens, Poland). A calibration curve was prepared using Trolox as the standard, and the results were expressed as µmol Trolox equivalents per 100 g of fresh weight (µmol TE 100 g^−1^ FW).

### 3.15. Statistical Analyses of the Results

The results were expressed as mean ± standard deviation. Data obtained after 6, 12, and 18 days of storage were analyzed separately. For each storage time, differences among experimental treatments were evaluated using one-way analysis of variance (ANOVA), followed by Tukey’s test at *p* < 0.05. Different lowercase letters indicate statistically significant differences among treatments within the same storage time. The initial sample analyzed before storage was presented descriptively and was not included in the statistical analysis. All statistical analyses were performed using Statistix 8.1 software.

## 4. Conclusions

The present study demonstrated that sachets containing biochar derived from spent tea residues may limit ethylene accumulation and promote the preservation of selected tomato quality attributes during storage. The effectiveness of the material generally depended on the mass of biochar applied. The treatment containing 10 g of biochar reduced the ethylene concentration in the cloche atmosphere by approximately 41.1%, 37.8%, and 40.3% relative to the control after 6, 12, and 18 days of storage, respectively.

The strongest effect on microbiological quality was obtained in the treatments containing 7.5 and 10 g of biochar, which showed the lowest counts of aerobic mesophilic microorganisms, yeasts, and molds. The application of at least 5 g of biochar also effectively limited firmness loss. After 18 days, the firmness of tomatoes stored with 5, 7.5, and 10 g of biochar was 1.77, 1.77, and 1.80 kgf, respectively, compared with 1.00 kgf in the control. Greater amounts of biochar also promoted the retention of titratable acidity, total phenolic content, and antioxidant activity measured using the ABTS and FRAP assays. After 18 days, the highest phenolic content was recorded in the 10 g treatment, at 53.46 mg GAE 100 g^−1^ FW, compared with 32.60 mg GAE 100 g^−1^ FW in the control. At the same storage time, ABTS activity in the 7.5 and 10 g treatments was 484.72 and 492.50 µmol TE 100 g^−1^ FW, respectively, whereas the control reached 330.19 µmol TE 100 g^−1^ FW. Visual observations also indicated less surface damage and better retention of tissue integrity than in the control. Biochar application had no significant effect on pH or total soluble solids.

The most favorable overall results were obtained with sachets containing 7.5 and 10 g of biochar. Spent-tea-leaf biochar may therefore represent a promising material for supporting ethylene control and preserving tomato quality in a closed storage system. This solution is consistent with circular economy and sustainable development principles because it combines the valorization of food-processing waste with the potential reduction in postharvest losses of fresh produce.

The results indicate that the use of spent-tea-leaf biochar in sachets may provide a basis for developing simple, passive, waste-derived systems that limit ethylene accumulation during fruit storage. To date, the literature has not presented a comprehensive evaluation of this type of material that simultaneously addresses its effects on ethylene concentration, microbiological quality, and the physicochemical and antioxidant properties of tomatoes during storage. Further studies should determine ethylene adsorption kinetics, include more frequent measurements during the initial storage period, particularly during the first 1–3 days, assess the safety of its use in systems intended for food-contact applications, and verify the effectiveness of the solution under practical packaging, transportation, and storage conditions. They should also include instrumental color measurements and quantitative assessment of decay incidence and marketable fruit rate.

## Figures and Tables

**Table 1:** Effect of different amounts of tea-waste biochar on total phenolic content and antioxidant activity of tomatoes after 6, 12, and 18 days of storage.

Parameter/Storage Period	Initial (Day 0)	Control	1 g	2 g	3 g	4 g	5 g	7.5 g	10 g
TPC (mg GAE 100 g^−1^ FW)									
Initial (day 0)	42.10 ± 0.63								
Day 6		44.83 ± 2.60 ^a^	42.18 ± 0.85 ^a^	44.74 ± 4.75 ^a^	41.13 ± 1.50 ^a^	42.19 ± 0.70 ^a^	42.89 ± 1.42 ^a^	46.40 ± 3.71 ^a^	44.16 ± 1.22 ^a^
Day 12		35.98 ± 1.45 ^c^	36.85 ± 2.34 ^c^	37.04 ± 0.97 ^c^	35.68 ± 1.29 ^c^	36.69 ± 0.97 ^c^	42.94 ± 0.91 ^b^	43.92 ± 1.08 ^b^	48.60 ± 1.15 ^a^
Day 18		32.60 ± 1.08 ^d^	32.09 ± 2.00 ^d^	32.64 ± 1.34 ^d^	33.14 ± 0.96 ^d^	37.15 ± 1.12 ^c^	48.85 ± 0.64 ^b^	48.92 ± 0.69 ^b^	53.46 ± 1.71 ^a^
ABTS (µmol TE 100 g^−1^ FW)									
Initial (day 0)	535.90 ± 13.08								
Day 6		496.77 ± 12.12 ^bc^	477.92 ± 11.66 c	480.44 ± 11.73 ^bc^	492.39 ± 12.02 ^bc^	495.89 ± 12.10 ^bc^	511.24 ± 12.48 ^bc^	513.98 ± 12.54 ^b^	565.27 ± 13.80 ^a^
Day 12		387.18 ± 9.45 ^d^	393.65 ± 9.61 ^cd^	420.61 ± 10.26 ^bc^	427.18 ± 10.43 ^b^	431.24 ± 10.52 ^b^	443.84 ± 10.83 ^b^	491.18 ± 11.99 ^a^	510.47 ± 12.46 ^a^
Day 18		330.19 ± 8.06 ^c^	336.33 ± 8.21 ^c^	339.29 ± 8.28 ^c^	353.65 ± 8.63 ^c^	406.69 ± 9.93 ^b^	398.80 ± 9.73 ^b^	484.72 ± 11.83 ^a^	492.50 ± 12.02 ^a^
DPPH (radical scavenging activity %)									
Initial (day 0)	85.56 ± 0.06								
Day 6		87.92 ± 0.03 ^a^	72.42 ± 8.63 ^b^	84.45 ± 2.53 ^ab^	77.70 ± 3.95 ^ab^	78.71 ± 7.33 ^ab^	77.42 ± 3.69 ^ab^	83.47 ± 2.61 ^ab^	85.12 ± 1.37 ^ab^
Day 12		67.66 ± 0.09 ^bc^	65.86 ± 0.66 ^c^	66.99 ± 4.61 ^c^	75.06 ± 0.01 ^abc^	70.06 ± 0.12 ^abc^	69.35 ± 8.84 ^abc^	77.60 ± 2.48 ^ab^	78.55 ± 2.01 ^a^
Day 18		61.85 ± 1.95 ^bc^	57.38 ± 0.12 ^c^	70.43 ± 8.68 ^ab^	61.25 ± 0.06 ^bc^	75.67 ± 0.03 ^a^	82.02 ± 1.24 ^a^	71.25 ± 8.79 ^ab^	80.00 ± 0.09 ^a^
FRAP (µmol TE 100 g^−1^ FW)									
Initial (day 0)	81.07 ± 0.95								
Day 6		91.56 ± 1.26 ^a^	87.10 ± 1.69 ^abc^	89.45 ± 3.04 ^ab^	81.76 ± 0.76 ^c^	84.40 ± 1.59 ^bc^	82.00 ± 2.13 ^c^	89.92 ± 4.00 ^ab^	88.56 ± 1.32 ^ab^
Day 12		70.56 ± 1.32 ^c^	66.52 ± 0.76 ^c^	69.84 ± 2.76 ^c^	67.16 ± 4.40 ^c^	72.24 ± 2.76 ^c^	82.92 ± 1.32 ^b^	94.60 ± 1.59 ^a^	95.44 ± 0.76 ^a^
Day 18		71.20 ± 0.76 ^b^	67.24 ± 1.59 ^bcd^	71.74 ± 2.49 ^b^	69.64 ± 2.13 ^bc^	65.08 ± 1.59 ^cd^	62.80 ± 1.76 ^d^	85.66 ± 1.39 ^a^	89.44 ± 3.82 ^a^

The initial values were determined before storage, are presented as reference values, and were not included in the statistical analysis. Different lowercase letters indicate statistically significant differences among treatments within the same storage period according to Tukey’s test (*p* < 0.05).

**Table 2:** Proximate composition, elemental composition and selected mineral element contents of biochar produced from leaf tea residues.

**Proximate and Elemental Composition (% Dry Matter)**			
**Parameter**		**Mean ± SD**	
Total carbon		85.39 ± 0.91	
Total nitrogen		4.80 ± 0.16	
Hydrogen		3.79 ± 0.02	
Ash		7.29 ± 0.03	
Volatile matter		30.65 ± 0.20	
**Mineral Element (mg 100 g^−1^ Dry Matter)**			
**Element**	**Mean ± SD**	**Element**	**Mean ± SD**
Ca	671.10 ± 14.90	Cd	0.005 ± 0.001
Cr	1.41 ± 0.09	Cu	1.48 ± 0.013
Fe	13.27 ± 1.21	K	548.20 ± 7.10
Mg	164.60 ± 2.70	Mn	37.51 ± 0.06
Mo	<LOQ	Na	32.81 ± 0.87
Ni	1.203 ± 0.008	P	1912.20 ± 15.50
Pb	0.03 ± 0.006	S	72.57 ± 0.61
Sr	2.11 ± 0.036	Zn	1.57 ± 0.004

## Data Availability

The original contributions presented in this study are included in the article. Further inquiries can be directed to the corresponding author.

## Abbreviations

The following abbreviations are used in this manuscript:

1-MCP	1-Methylcyclopropene
ABTS	2,2′-Azino-bis(3-ethylbenzothiazoline-6-sulfonic acid)
AOAC	AOAC International
CFU	Colony-forming units
DPPH	2,2-Diphenyl-1-picrylhydrazyl
FRAP	Ferric reducing antioxidant power
FW	Fresh weight
GAE	Gallic acid equivalents
ICP-OES	Inductively coupled plasma optical emission spectrometry
ISO	International Organization for Standardization
LOQ	Limit of quantification
PR	Pathogenesis-related
SD	Standard deviation
SDG	Sustainable Development Goal
TE	Trolox equivalents
TPC	Total phenolic content
TPTZ	2,4,6-Tris(2-pyridyl)-s-triazine
TSS	Total soluble solids
UV–Vis	Ultraviolet–visible spectroscopy
